# Identification of the Hypoglycemic Active Components of *Lonicera japonica* Thunb. and *Lonicera hypoglauca* Miq. by UPLC-Q-TOF-MS

**DOI:** 10.3390/molecules29204848

**Published:** 2024-10-13

**Authors:** Qinxuan Wu, Di Zhao, Ying Leng, Canhui Chen, Kunyu Xiao, Zhaoquan Wu, Fengming Chen

**Affiliations:** 1 Hunan Provincial Key Laboratory of the Traditional Chinese Medicine Agricultural Biogenomics, The “Double-First Class” Application Characteristic Discipline of Hunan Province (Pharmaceutical Science), Changsha Medical University, Changsha 410219, China; qinxuanwucsmu@163.com (Q.W.); didixiao0806@163.com (D.Z.); csyxy613@163.com (C.C.); xiaokunyuxiaokunyu@163.com (K.X.); wuzq900815@163.com (Z.W.); 2 Hunan Pharmaceutical Development and investment Group Co., Ltd., Changsha 410219, China; liangjl3354@163.com

**Keywords:** *Lonicera japonica* Thunb., *Lonicera hypoglauca* Miq., hypoglycemic activity, UPLC-Q-TOF-MS, isochlorogenic acid A, isochlorogenic acid C

## Abstract

*Lonicera japonica* Thunb. and *Lonicera hypoglauca* are famous Chinese medicines used for hyperglycemia; however, the specific compounds that contributed to the hypoglycemic activity and mechanism are still unknown. In this study, the antidiabetic activity of *L. japonica* buds and *L. hypoglauca* buds, roots, stems, and leaves extracts was primarily evaluated, and the *L. japonica* buds and *L. hypoglauca* buds, roots, and stems extracts displayed significant hypoglycemic activity, especially for the buds of *L. hypoglauca*. A total of 72 high-level compounds, including 9 iridoid glycosides, 12 flavonoids, 34 organic acids, and 17 saponins, were identified by ultra-performance liquid chromatography/quadrupole time-of-flight mass spectrometry (UPLC-Q-TOF-MS) combined with the fragmentation pathways of standards from different parts of *L. japonica* and *L. hypoglauca* extracts. Among them, 19 metabolites, including 13 saponins, were reported for the first time from both medicines. Seven high-content compounds identified from *L. hypoglauca* buds extract were further evaluated for hypoglycemic activity. The result indicated that neochlorogenic acid, chlorogenic acid, isochlorogenic acid A, isochlorogenic acid B, and isochlorogenic acid C displayed significant antidiabetic activity, especially for isochlorogenic acid A and isochlorogenic acid C, which demonstrated that the five chlorogenic-acid-type compounds were the active ingredients of hypoglycemic activity for *L. japonica* and *L. hypoglauca*. The potential mechanism of hypoglycemic activity for isochlorogenic acid A and isochlorogenic acid C was inhibiting the intestinal α-glucosidase activity to block the supply of glucose. This study was the first to clarify the hypoglycemic active ingredients and potential mechanism of *L. japonica* and *L. hypoglauca*, providing new insights for the comprehensive utilization of both resources and the development of hypoglycemic drugs.

## 1. Introduction

*Lonicera japonica* Thunb. and *Lonicera hypoglauca* Miq., which belong to the genus of Lonicaceae from Caprifoliaceae family, are mainly distributed in Asia, North America, Europe, and temperate or subtropical regions of northern Africa [1,2]. At present, *L*. *japonica* is mainly planted in Shandong, Henan, Hebei province, and other areas north of the Yangtze River in China, while *L. hypoglauca* is distributed in the south of the Yangtze River in China, such as in Hunan and Zhejiang provinces [3,4]. *L. japonica* and *L. hypoglauca* are included in the 2020 edition of the Chinese Pharmacopoeia and have the effects of clearing heat and detoxifying, and they are used for the treatment of wind-heat cold, fever, heat, blood dysentery, and other diseases [5,6]. Modern pharmacological studies have proved that both Chinese medicines have antiviral, antibacterial, immune-modulating, liver-protecting, antipyretic, antitumor, neuroprotective, and hypoglycemic effects [7,8]. *L. japonicae* has antiplatelet aggregation, glucose-lowering, antiulcer, antiultraviolet radiation, antiendotoxin, antifertility, anti-early pregnancy, and neuroprotective activities that are not reported in *L. hypoglauca*. In addition, *L. hypoglauca* has a certain advantage in terms of antibacterial activity compared to that of *L. japonicae* [1,4]. The main active ingredients are organic acids, flavonoids, iridoids, and saponins [9,10]. *L. japonicae* contains more flavonoids and iridoids; however, *L. japonicae* have a variety of saponins and significantly higher chlorogenic acid than *L. japonicae* [1,4]. In addition to the well-known components, there are many chemical components present in *L. japonica* and *L. hypoglauca* that need to be further studied and identified.

During plant growth, its metabolites are constantly changing, and the metabolites in the buds, roots, stems, and leaves of *L. japonica* and *L. hypoglauca* also vary [11,12]. In previous studies, liquid chromatography–mass spectrometry (LC-MS) was used to identify the chemical composition of a single part of *L. japonica* or *L. hypoglauca* [13,14,15], resulting in the loss of a portion of the compound. Therefore, in this study, we comprehensively analyzed the chemical composition in the buds, roots, stems, and leaves of *L. japonica* and *L. hypoglauca* by UPLC-Q-TOF-MS technology, laying the foundation for the subsequent identification of hypoglycemic active substances.

Diabetes is currently one of the most common metabolic diseases and has a significant impact on human health worldwide [16,17]. According to the International Diabetes Federation (2019), the number of people with diabetes is expected to reach 578 million in 2030, and type 2 diabetes accounts for about 90% of the total [18,19]. However, synthetic drugs to treat type 2 diabetes cause many adverse effects, and traditional Chinese herbal medicines or natural ingredients have received extensive attention in many countries due to their significant antidiabetic activity and low adverse reactions [20,21]. Therefore, the development of new antidiabetic drugs from traditional herbal medicines has been a hot topic of research. In previous studies [22,23,24,25], it has been reported that *L. japonica* polysaccharides have significant hypoglycemic activity but there are few studies on the hypoglycemic activity of *L. hypoglauca.* Moreover, the specific hypoglycemic components of *L. japonica* or *L. hypoglauca* are still not clear.

In this study, we first evaluated the hypoglycemic activity of extracts from *L. japonica* and different parts of *L. japonica*; then, UPLC-Q-TOF-MS technology was employed to identify their potential hypoglycemic components in different parts of both medicines, and seven monomeric compounds with high content in *L. japonica* and *L. hypoglauca* were finally used to clarify the hypoglycemic components of both Chinese medicines and the potential mechanism.

## 2. Results and Discussion

### 2.1. Evaluation of the Hypoglycemic Activity of L. japonica and Different Parts of L. hypoglauca Extract

**Figure 1:**
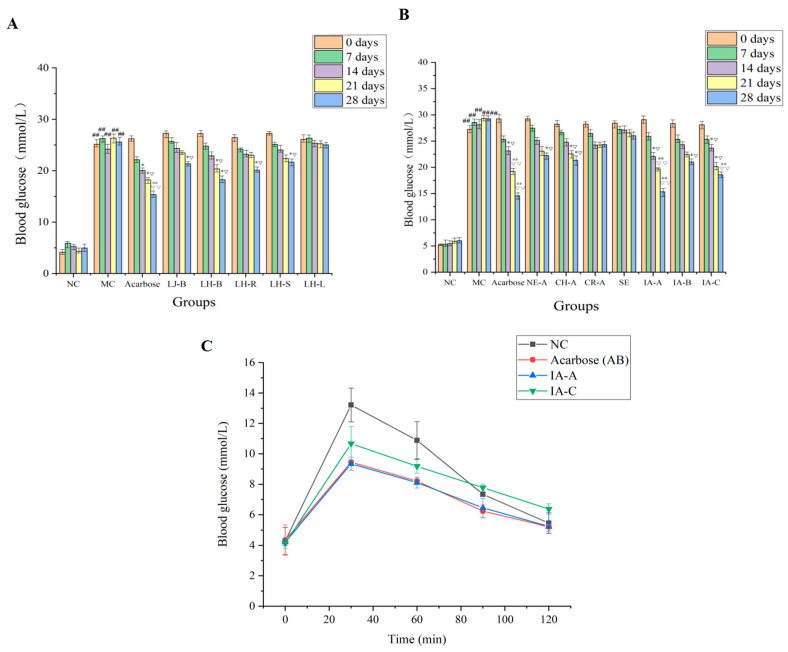
Effect of *L. japonica* buds, *L. hypoglauca* buds, roots, stems, leaves extract, and monomeric compounds on blood glucose concentrations. (**A**) The hypoglycemic activity of *L. japonica* buds, *L. hypoglauca* buds, roots, stems, and leaves extracts; (**B**) the hypoglycemic activity of 7 high-content compounds from the extracts; (**C**) the postprandial glycemia of administration of isochlorogenic acid A (IA-A) and isochlorogenic acid C (IA-C) for the normal mice. ^##^ *p* < 0.01 compared with the normal control group on the same days. * *p* < 0.05 or ** *p* < 0.01 compared with the data at day 0; ^▽^ *p* < 0.05 or ^▽▽^ *p* < 0.01 compared with the model control group on the same days. NC: normal group; MC: model group; Acarbose group (positive control, 30 mg/kg); LJ-B: *L. japonica* buds group (200 mg/kg); LH-B: *L. hypoglauca* buds group (200 mg/kg); LH-R: *L. hypoglauca* roots group (200 mg/kg); LH-S: *L. hypoglauca* stems group (200 mg/kg); LH-L: *L. hypoglauca* leaves group (200 mg/kg); NE-A: neochlorogenic acid group (30 mg/kg); CH-A: chlorogenic acid group (30 mg/kg); CR-A: cryptochlorogenic acid group (30 mg/kg); SE: secoxyloganin group (30 mg/kg); IA-A: isochlorogenic acid A group (30 mg/kg); IA-B: isochlorogenic acid B group (30 mg/kg); and IA-C: isochlorogenic acid C group (30 mg/kg).

*L. japonica* have been reported to have significant hypoglycemic activity [22,23,24,25], while *L. hypoglauca* extracts and other components present in both medicines have rarely been reported to have antidiabetic activity. To evaluate the hypoglycemic activity of non-polysaccharides in *L. japonica* bud and *L. hypoglauca* buds, roots, stem, and leaves, 70% ethanol was employed as the solvent to obtain the corresponding extracts. The result indicated that the blood glucose level of the model control group (MC) was significantly higher (*p* < 0.01) compared with the normal control group (NC) at day 0, 7, 14, 21, and 28, respectively, which indicated that the high blood glucose mouse model (T2D) was successfully established (Figure 1A). The blood glucose level of the acarbose group at day 14 (20.01 ± 0.33 mmol/L), 21 (18.21 ± 0.28 mmol/L), and 28 (15.34 ± 0.21 mmol/L) significantly decreased (*p* < 0.05) compared with the initial level at day 0 (26.20 ± 0.54 mmol/L), which indicated that the positive group displays significant hypoglycemic activity. The blood glucose level of *L. japonica* bud extract at day 28 (21.34 ± 1.09 mmol/L) in the groups was significantly decreased (*p* < 0.05) compared with the initial blood glucose level at day 0 (27.26 ± 0.45 mmol/L), which indicated that the *L. japonica* buds extracts have remarkable hypoglycemic activity (Figure 1A). Compared with the initial blood glucose level at day 0 (27.21 ± 0.45, 26.45 ± 1.21, and 27.23 ± 0.23 mmol/L), the buds, roots, and stems extracts (18.22 ± 1.82, 20.11 ± 1.23, and 21.67 ± 1.02 mmol/L, respectively) groups at day 28 were significantly decreased (*p* < 0.05), which indicated that the *L. hypoglauca* buds, roots, and stem extracts display significant antidiabetic activity, especially for the extract of buds. However, the leaves extract did not show significant activity in decreasing blood glucose level.

### 2.2. Identification of the Main Components of L. japonica and Different Parts of L. hypoglauca Extracts

The 70% ethanol extracts of *L. japonica* buds and *L. hypoglauca* buds, roots, and stems had significant hypoglycemic activity, but the specific components that contributed to the hypoglycemic activity were still unknown. In this study, the chemical components in the 70% ethanol extracts were further systematically identified by the UPLC-Q-TOF-MS technology, and the potential hypoglycemic active components in the 70% ethanol extract were proposed.

**Figure 2:**
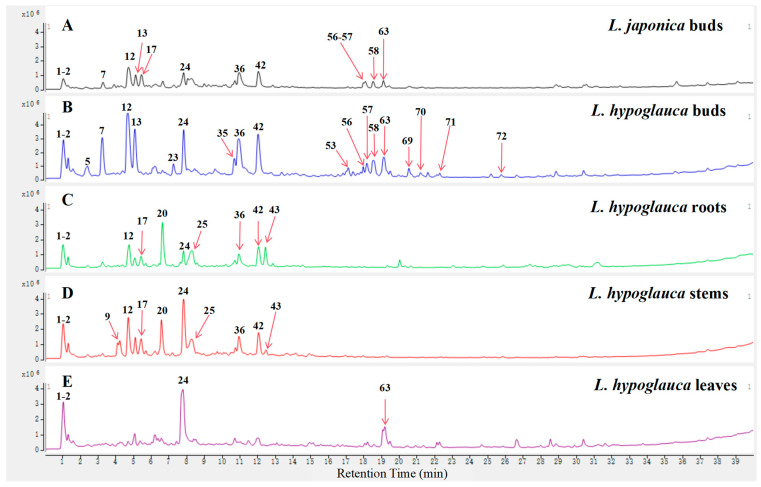
The TICs of *L. japonica* buds (**A**), *L. hypoglauca* buds (**B**), roots (**C**), stems (**D**), and leaves (**E**).

**Figure 3:**
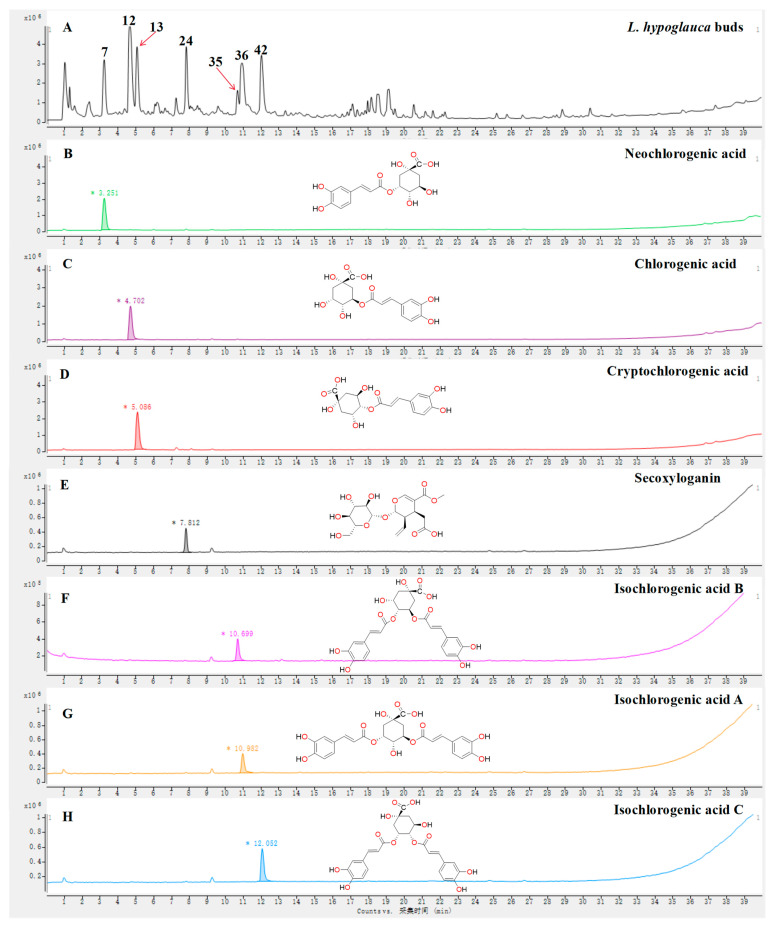
The TICs of *L. japonica* buds (**A**), neochlorogenic acid (**B**), chlorogenic acid (**C**), cryptochlorogenic acid (**D**), secoxyloganin (**E**), isochlorogenic acid B (**F**), isochlorogenic acid A (**G**), and isochlorogenic acid C (**H**).

The total ion chromatograms (TICs) of *L. japonica* bud and *L. hypoglauca* buds, roots, stems, and leaves (Figure 2) were primarily produced using UPLC-Q-TOF-MS technologies. And, then, the MS/MS spectra of metabolites were obtained by the target MS/MS method or auto MS/MS strategy. Finally, a total of 72 metabolites (Table 1), which may contribute to the hypoglycemic activity, were screened and identified by their MS/MS spectra combined with the well-investigated fragmentation pathways of standards. The structures of high-level metabolites **7**, **12**, **13**, **24**, **35**, **36**, and **42** were unambiguously determined by comparing the retention time, MS, and MS/MS data with the references (Figure 3). The other compounds were tentatively identified by the established UPLC-Q-TOF-MS method.

#### 2.2.1. Investigation of the Fragmentation Behaviors of Standards

A series of analogs are presented in *L. japonica* buds and *L. hypoglauca* buds, roots, stems, and leaves. Investigating the fragmentation behaviors of well-characterized standards is a valid strategy for identifying the unknown structures of analogs since they typically display similar fragmentation patterns in the MS/MS spectra. In this study, a total of seven standards, including neochlorogenic acid (**7**), chlorogenic acid (**12**), cryptochlorogenic acid (**13**), secoxyloganin (**24**), isochlorogenic acid B (**35**), isochlorogenic acid A (**36**), and isochlorogenic acid C (**42**), which were reported from *L. japonica* or *L. hypoglauca* in previous studies [4], were employed for investigating the fragmentation pathways of organic acid and iridoid glycosides. The fragmentation pathways of flavonoids and saponins in both medicines were also summarized based on previous studies [11,26].

**Figure 4:**
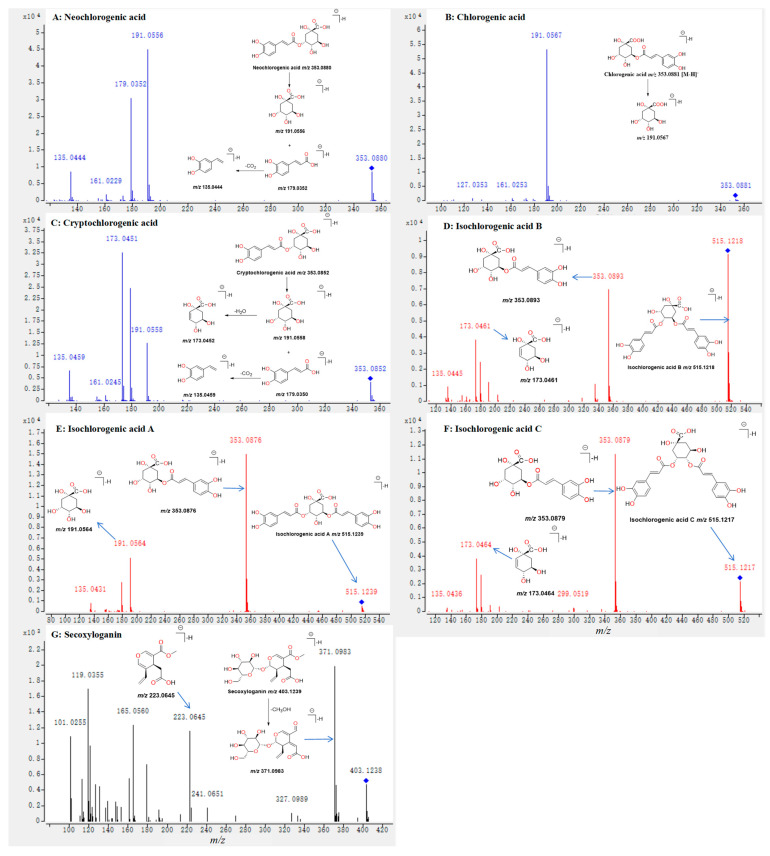
The MS/MS spectra of neochlorogenic acid (**A**), chlorogenic acid (**B**), cryptochlorogenic acid (**C**), isochlorogenic acid B (**D**), isochlorogenic acid A (**E**), isochlorogenic acid C (**F**), and secoxyloganin (**G**), and the corresponding characteristic ions and fragmentation pathways.

The fragmentation pathways of chlorogenic-acid-type in *L. japonica* or *L. hypoglauca* were investigated from the MS/MS spectra of standards **7**, **12**, **13**, **35**, **36**, and **42**. Two main fragmentation behaviors were observed. The primary fragmentation pathway was the cleavage of the bond between the quinic acid and caffeic acid and the formation of the high-abundance ions. In the MS/MS of **7**, **12**, **13**, **35**, **36**, and **42** (Figure 4A–F), the characteristic ions at *m*/*z* 353.08, 191.05, and 179.03 were produced by the cleavage of the bond between the quinic acid and caffeic acid. The other fragmentation pathway was the loss of some molecules, such as H_2_O and CO_2_, from the fragment ions. In the MS/MS of **13**, **35**, and **42**, the ions at *m*/*z* 173.0451, 173.0461, and 173.0464 were formed by the neutral loss of a H_2_O moiety from the fragments at *m*/*z* 191.0558, 191.0560, and 151.0556, respectively. Two fragmentation behaviors were found for iridoid glycosides. The first fragmentation pathway was the loss of some neutral moiety, such as CH_3_OH and CO_2_, from the fragment ions and the formation of characteristic ions. In the MS/MS of **24** (Figure 4G), the ion at *m*/*z* 371.0983 was produced by the loss of a CH_3_OH from the ion at *m*/*z* 403.1238. The second fragmentation pathway was the loss of the sugar moiety. In the MS/MS of **24** (Figure 4G), the ion at *m*/*z* 223.0645 was obverse due to the loss of the sugar moiety from the ion at *m*/*z* 403.1238. Three fragmentation pathways of flavonoids present in *L. japonica* and *L. hypoglauca* have been well concluded in previous studies [24]. The loss of all sugar moiety from the mother ion and the form of the characteristic ion at *m*/*z* 323.09 was the main fragmentation pathway of saponins present in both medicines [25].

#### 2.2.2. Identification of the Iridoid Glycosides, Flavonoids, Organic Acid, and Saponins

**Figure 5:**
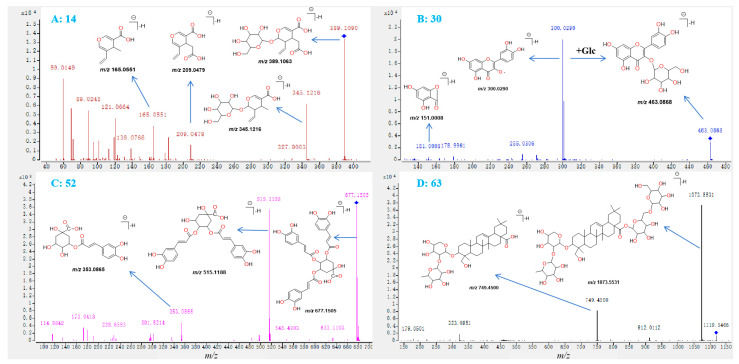
The identification of compounds **14** (**A**), **30** (**B**), **52** (**C**), and **63** (**D**) according to their MS/MS spectra and the corresponding characteristic fragment ions.

A total of nine compounds, including compounds **3**, **9**, **14**, **15**, **17**, **20**, **24**, **28**, and **43**, were identified as iridoid glycosides based on their MS/MS spectra (Table 1 and Figure S1). Take compound **14** as an example. The MS fragmentation pathways of compounds **14** and **42** (standard) were highly similar (Figure 4G and Figure 5A), and the difference in *m*/*z* value between both compounds was 14.0148 Da, which indicated that the methyl group in compound **42** was replaced by a hydrogen atom and formed the structure of metabolite **14**. Therefore, compound **14** was tentatively identified as Secologanoside-7-methyl ester, which has been reported in a previous study [4]. Compounds **25**, **26**, **29**–**34**, **37**–**39**, and **45** were identified as flavonoids based on their characteristic MS/MS spectra. In the ms/ms spectrum of compound **30** (Figure 5B), the loss of a glucose moiety from the mother ion at *m*/*z* 463.0868 and the formation of the fragment ion at *m*/*z* 300.0290 was found, which indicated that the skeleton of compound **30** was quercetin and a glucose moiety was concluded in the structure; therefore, compound **30** was tentative as hyperoside, which was also reported in a previous study [4]. A total of 34 metabolites, including **1**, **2**, **4**, **5**, **6**–**8**, **10**–**13**, **16**, **18**, **19**, **21**–**23**, **27**, **35**, **36**, **40**–**42**, **44**, **46**, **47**–**52**, **60**, **62**, and **68,** were identified as organic acid based on their MS/MS spectra (Table 1 and Figure S1 in the Supplementary Materials). Take compound **52** as an example. The MS fragmentation pathways of compounds **52** and **35** were highly similar (Figure 4D and Figure 5C) and the difference in *m*/*z* value between both compounds was 162.0287 Da, which indicated that one caffeic acid was added to compound **35** and formed the structure of metabolite **52**. The characteristic ions at *m*/*z* 515.1188, 353.0865, and 173.0418 indicated that compound 52 was 3,4,5-tricaffeoylquinic acid [4]. Compounds **53**–**59**, **61**, **63**–**67**, and **69**–**72** were identified as saponins based on their characteristic fragment ions. In the MS/MS spectrum of compound **63** (Figure 5D), the characteristic fragment ions at *m*/*z* 1073.5531, 749.4500, and 323.0951 indicated that compound **30** was dipsacoside B, which was also reported in a previous study [4].

Finally, a total of 72 metabolites, including 9 iridoid glycosides, 12 flavonoids, 34 organic acids, and 17 saponins, were identified using their MS/MS spectra, fragmentation pathways of standards, and the previous studies. Among these, 19 compounds (**20**, **28**, **44**, **48**, **49**, **53**–**56**, **58**, **60**, **61**, **65**, and **67**–**72**) including 13 saponins were reported for the first time from both medicines (Table 1). The primary metabolites of *L. japonica* bud and *L. hypoglauca* buds, roots, stems, and leaves, such as compounds **7**, **12**, **13**, **24**, **35**, **36**, **42**, **58**, and **63**, were well identified in this study, which may contribute to the antidiabetic activity of the extracts.

The distribution of all identified metabolites in the *L. japonica* bud and *L. hypoglauca* buds, roots, stems, and leaves was determined using the extracted ion chromatogram (EIC) based on the TICs. The identified compounds were found in all parts; however, the relative content of specific compounds in different parts has significant differences. The species and amount of metabolites in the *L. hypoglauca* buds were more abundant than in other parts, especially for the organic acids and saponins. Most of the high-level organic acids and saponins, such as compounds **7**, **12**, **13**, **36**, **42**, **58**, and **63**, were primarily detected in *L. hypoglauca* buds. The identified iridoid glycosides, such as compounds **17** and **20**, are mainly distributed in the roots and stems of *L. hypoglauca*. The main components of *L. hypoglauca* leaves were secoxyloganin (**24**) and dipsacoside B (**63**) and the level of other metabolites was relatively low.

### 2.3. Evaluation of the Hypoglycemic Activity of Seven High-Content Compounds in L. japonica and L. hypoglauca Extracts

*L. japonica* buds and *L. hypoglauca* buds, roots, and stem extracts display significant antidiabetic activity, especially for the extract of *L. hypoglauca* buds. However, the specific compounds that contributed to the hypoglycemic activity are still unknown. UPLC-Q-TOF-MS analysis results indicated that compounds **7** (neochlorogenic acid), **12** (chlorogenic acid), **13** (cryptochlorogenic acid), **24** (secoxyloganin), **35** (isochlorogenic acid B), **36** (isochlorogenic acid A), and **42** (isochlorogenic acid C) were the main components of *L. hypoglauca* bud extract. To clarify the hypoglycemic active ingredients of *L. japonica* and *L. hypoglauca*, the antidiabetic activity of seven high-content compounds were evaluated.

The blood glucose level of the model control group (MC) was significantly higher (*p* < 0.01) compared with the normal control group (NC) at day 0, 7, 14, 21, and 28, respectively, which indicated that the high blood glucose model was successfully established (Figure 1B). The blood glucose level of the acarbose group at day 14 (23.11 ± 1.33 mmol/L), 21 (19.21 ± 1.28 mmol/L), and 28 (14.56 ± 1.21 mmol/L) significantly decreased (*p* < 0.05) compared with the initial level at day 0 (29.23 ± 1.52 mmol/L), which indicated that the positive group displays significant hypoglycemic activity. Compared with the initial blood glucose level at day 0 (29.23 ± 0.67, 28.23 ± 1.22, and 28.24 ± 1.45 mmol/L for NE-A, CH-A, and CR-A, respectively), the NE-A (22.23 ±1.29 mmol/L) and CH-A (21.34 ± 1.82 mmol/L) groups at day 28 were significantly decreased (*p* < 0.05), which indicated that the neochlorogenic acid (**7**) and chlorogenic acid (**12**) have remarkable hypoglycemic activity (Figure 1B); however, the cryptochlorogenic acid (**13**) did not show significant activity. The blood glucose level of the IA-A and IA-C at day 14 (22.08 ± 1.08 and 23.67 ± 1.18 mmol/L), 21 (19.67 ± 0.56 and 20.12 ± 1.32 mmol/L), and 28 (15.34 ± 1.21 and 18.56 ± 1.07 mmol/L) significantly decreased (*p* < 0.05 or *p* < 0.01) compared with the initial level at day 0 (29.09 ± 1.17 and 28.11 ± 1.27 mmol/L), which indicated that the isochlorogenic acid A (**36**) and isochlorogenic acid C (**42**) have significant hypoglycemic activity and the antidiabetic activity of isochlorogenic acid A was stronger than that of isochlorogenic acid C. In addition, IA-B (**35**) also displays significant antidiabetic activity, which could decrease the blood glucose level from 28.34 ± 1.24 mmol/L (day 0) to 21.01 ± 1.42 mmol/L (day 28); however, its hypoglycemic activity was weaker than that of isochlorogenic acid A and isochlorogenic acid C.

### 2.4. The Effect of Isochlorogenic Acid A (**36**) and Isochlorogenic Acid C (**42**) on the Postprandial Glycemia

Isochlorogenic acid A (**36**) and isochlorogenic acid C (**42**), which were the most active compounds, were employed to explore the potential mechanism of hypoglycemic activity. The postprandial glycemia experiment for both compounds was carried out. The result shows that the acarbose (9.45 ± 0.2 mmol/L), isochlorogenic acid A (9.34 ± 0.43 mmol/L), and isochlorogenic acid C (10.67 ± 1.12 mmol/L) groups display significant activity in reducing postprandial glycemia compared to the normal control group (13.21 ± 1.11 mmol/L) after administration of corresponding solution for 30 min (Figure 1C). The effect on postprandial glycemia of isochlorogenic acid A was the same as the positive drug (acarbose). Isochlorogenic acid A displays stronger activity than isochlorogenic acid C for reducing postprandial glycemia. The blood glucose level of all groups returns to baseline after 120 min. The above results indicate that isochlorogenic acid A, isochlorogenic acid C, *L. japonica* buds, *L. hypoglauca* buds, roots, and stem extracts inhibiting the intestinal α-glucosidase activity to block the supply of glucose was the potential mechanism of hypoglycemic activity.

## 3. Materials and Methods

### 3.1. Materials and Chemicals

Ethanol (AR) was purchased from Chengdu Must Bio-Technology Co., Ltd. (Chengdu, China), which was used for extraction. Acetonitrile (HPLC-grade) was obtained from Merck (Darmstadt, Germany). Acarbose (purity ≥ 98%), streptozotocin (STZ), and seven standards, including neochlorogenic acid (**7**), chlorogenic acid (**13**), cryptochlorogenic acid (**14**), secoxyloganin (**24**), isochlorogenic acid B (**35**), isochlorogenic acid A (**36**), and isochlorogenic acid C (**42**), were obtained from National Institutes for Food and Drug Control (Beijing, China). The high-fat feed (MD12033, 60% fat kcal%) was purchased from Jiangsu Medicience Biomedical Pharmaceuticals Limited; for the specific components of high-fat feed, see Table S1.

### 3.2. Extracts Experiment

*L. hypoglauca* buds, roots, stems, and leaves were collected in September of 2023 at the Hunan Agricultural University (Hunan, China, co-ordinates: 113°04′25.55″ E, 28°10′45.11″ N). They were authenticated by Prof. Han Zhou (Hunan Agricultural University, China). The fresh buds, roots, stems, and leaves were dried under a vacuum drying oven at 60 °C for 48 h. The *L. japonica* buds were purchased from LBX pharmacy (Changsha, China) and both dried samples were crushed by a disintegrator. Approximately 50.0 g of dried sample powder was suspended in 500 mL of 70% ethanol water (*v*/*v*) for 30 min. Extraction was carried out in an ultrasonic bath for 60 min, and the extract solution was filtered through a nylon membrane. *L. japonica* buds and *L. hypoglauca* buds, roots, stems, and leaves extracts were obtained after recovering the solvent by vacuum reduction concentration.

### 3.3. UPLC-Q-TOF-MS Conditions

An Agilent 1290 UPLC system coupled with a 6530 Q-TOF/MS accurate mass spectrometer (Agilent Technologies Inc., Santa Clara, CA, USA) was used for the investigation of the fragmentation pathways of standards and identification of compounds in *L. japonica* and *L. hypoglauca*. An Agilent C18 (150 mm × 2.1 mm, 1.7 µm; Agilent Technologies Inc., Santa Clara, CA, USA) was used as a separation column. The elution solution consisted of 0.1% formic acid–water (A) and 0.1% formic acid–acetonitrile (B). The elution system was 0–15 min, 5–40% B; 15–25 min 40–65% B; and 25–40 min 65–90% B. The flow rate was set at 0.30 mL/min and the injection volume was 3 μL. The conditions of Q-TOF-MS (negative mode) were optimized as follows: gas temperature and sheath gas temperature were set at 350 and 300 °C, respectively. Sheath gas and drying gas flow were optimized to 10 and 11 L/min, respectively. The fragment voltage was set at 150 V. The Q-TOF-MS was continuously calibrated by a reference solution at *m*/*z* 112.9855 and 966.0007. The MS/MS data of each compound were obtained using the target-MS/MS mode (10–25 eV) and auto-MS/MS mode (15, 20, and 30 eV).

### 3.4. Experimental Animal

The male ICR mice, which were four weeks old with a weight range of 18.0–22.0 g, were purchased from Hunan Slake Jing-da Experimental Animals Co., Ltd. (Certificate number 43004700048590, Changsha, China). Animals were kept under the ambient temperature of 22 ± 2 °C, and a standard light/dark schedule (12:12 h) was used for those mice. All experiments and procedures were carried out based on the Regulations of Experimental Animal Administration issued by the State Committee of Science and Technology of China.

### 3.5. Establishment of High-Glucose Mouse Model (T2D) and the Drug Administration

The mice were subjected to a high-fat diet for 30 days in addition to the normal control group (NC). After the high-fat diet feeding, the T2D mouse model was successfully induced by intraperitoneal injection of STZ for 3 days (60 mg/kg) when the blood glucose level was more than 11.6 mmol/L. The normal and model control groups were given distilled water and the rest of the groups were given the corresponding solution of extracts or drugs by intragastric administration (200 mg/kg for the extracts and 30 mg/kg for acarbose and 7 standards, respectively) with a volume of 20 mL/kg (Tables S2 and S3). Mice were treated daily for a continual 28 days. The blood glucose level was determined by a glucometer (Johnson, Branchburg, NJ, USA) for every 7-day interval of 28 days.

### 3.6. The Experiment on Postprandial Glycemia of the Normal Mice

Forty normal mice, which were four weeks old with a weight range of 18.0–22.0 g, were randomly divided into four groups based on their weight, each containing ten mice. All mice were given a sucrose solution with 2.0 g/kg. In addition to the normal control group (NC), the remaining three groups were given acarbose (30 mg/kg), isochlorogenic acid A (**36**), and isochlorogenic acid C (**42**) solution (30 mg/kg). The blood glucose level was determined by the glucometer at 0, 30, 60, 90, and 120 min.

### 3.7. Statistical Method

The experimental data statistical analysis was carried out using SPSS16.0, and the statistically significant level was set as *p* ≤ 0.05. The data were expressed as mean ± standard deviation. The normality and variance homogeneity were tested by Leven’s test method, and the statistical analysis was performed with a one-way analysis of variance (ANOVA) and LSD test if the normality and variance homogeneity were met (*p* > 0.05); otherwise, the Kruskal–Wallis test was used. If the Kruskal–Wallis test was statistically significant (*p* ≤ 0.05), the Dunnett’s test (nonparametric method) was used for comparative analysis. Statistical differences and biological significance were considered simultaneously in this study.

## 4. Conclusions

In this study, the antidiabetic activity of *L. japonica* buds, *L. hypoglauca* buds, roots, stems, and leaves extracts was primarily evaluated, and the *L. japonica* buds, *L. hypoglauca* buds, roots, and stems extracts displayed significant hypoglycemic activity, especially for the buds of *L. hypoglauca*. Then, a total of 72 high-level metabolites were screened and identified from the *L. japonica* buds, *L. hypoglauca* buds, roots, stems, and leaves extracts by UPLC-Q-TOF-MS combined with the fragmentation behaviors of references. Among these, 19 compounds were reported for the first time from both medicines. Five high-content compounds, including neochlorogenic acid, chlorogenic acid, isochlorogenic acid A, isochlorogenic acid B, and isochlorogenic acid C, displayed significant hypoglycemic activity, especially for isochlorogenic acid A, which demonstrated that these compounds contributed to the hypoglycemic activity of *L. japonica* and *L. hypoglauca*. The potential mechanism of hypoglycemic activity for both medicines or the active compounds was inhibiting the intestinal α-glucosidase activity to block the supply of glucose. However, the inhibitory activity of α-glucosidase in vitro should be further investigated to further clarify their hypoglycemic mechanism. This study was the first to clarify the hypoglycemic active ingredients of *L. japonica* and *L. hypoglauca*, which provides new insights for the comprehensive utilization of both resources and the development of new hypoglycemic drugs.

## Figures and Tables

**Table 1:** The identified compounds from *L. japonica* and *L. hypoglauca* by UPLC-Q-TOF-MS method.

No.	*t*_R_ (min.)	(*m*/*z*)	Error (ppm)	Molecular Formula	Belongs	MS/MS Fragment Ions (*m*/*z*)	Identification
1	0.96	191.0544	−3.1	C_7_H_12_O_6_	All	173.0421, 127.0396, 109.0302	Citric acid
2	1.04	191.0553	−3.6	C_7_H_12_O_6_	All	173.0452, 127.0388	Quinic acid
3	1.31	387.1305	−4.9	C_17_H_24_O_10_	All	341.1096, 179.0579, 119.0388	Secologanin
4	1.61	161.0448	−1.2	C_6_H_10_O_5_	All	101.0170, 57.0362	Meglutol
5	2.46	329.0874	2.1	C_14_H_18_O_9_	All	167.0344, 152.0089, 108.0231	Phaseoloidin
6	2.54	218.1029	3.2	C_9_H_17_NO_5_	All	146.0801, 125.0219, 116.0684	Pantothenic acid
7 *^c^*	3.25	353.0882	2.5	C_16_H_18_O_9_	All	191.0565, 179.0352, 135.0456	Neochlorogenic acid
8	4.09	299.0770	1.0	C_13_H_16_O_8_	All	137.0244, 101.0247	Protamin sulfate- 4-glucoside
9	4.11	375.1304	4.8	C_16_H_24_O_10_	All	213.0779, 179.0509, 169.0840	Loganic acid
10	4.41	175.0598	−1.1	C_7_H_12_O_5_	All	157.0569, 115.0420, 113.0620	Isopropylmalic acid
11	4.61	329.0860	−2.1	C_14_H_18_O_9_	All	167.0356	Isophaseoloidin
12 *^c^*	4.70	353.0872	−0.2	C_16_H_18_O_9_	All	191.0560, 179.0351, 135.0454	Chlorogenic acid
13 *^c^*	5.08	353.0878	1.4	C_16_H_18_O_9_	All	191.0560, 179.0351, 135.0454	Cryptochlorogenic acid
14	5.10	389.1090	3.0	C_16_H_22_O_11_	All	345.1191, 209.0425, 165.0551	Secologanoside- 7-methyl ester
15	5.15	389.1063	3.8	C_16_H_22_O_11_	All	345.1205, 209.0440, 183.0642, 165.0545	Secologanoside
16	5.31	179.0332	−3.9	C_9_H_8_O_4_	All	135.0453, 107.0505	Caffeic acid
17	5.34	373.1098	−8.3	C_16_H_22_O_10_	All	193.0492, 179.0563, 149.0586	Swertiamarine
18	6.24	187.0976	2.6	C_9_H_16_O_4_	All	125.0900, 97.0657	Azelaic Acid
19	6.28	337.0922	−0.2	C_16_H_18_O_9_	All	191.0554, 173.0436, 163.0397	5-*O*-p-coumaroylquinic acid
20 ^*a*^	6.51	435.1545	9.6	C_17_H_26_O_10_	All	227.0935, 165.0539, 101.0252	Dihydrogen-vogeloside
21	6.61	153.0176	−3.9	C_7_H_6_O_4_	All	135.0092, 109.0281	Protocatechuic acid
22	6.85	153.0186	2.6	C_7_H_6_O_4_	All	135.0028, 109.0261	Hypogallic acid
23	7.24	367.1020	2.4	C_17_H_20_O_9_	All	193.0503, 191.0550, 173.0439	Methyl-chlorogenic acid
24 *^c^*	7.81	403.1239	−0.2	C_17_H_24_O_11_	All	371.0983, 223.0636, 179.0606, 165.0566, 121.0303	Secoxyloganin
25	8.29	609.1471	2.2	C_27_H_30_O_16_	All	301.0343, 300.0247	Lutin
26	8.38	595.1269	−5.0	C_26_H_28_O_16_	All	301.0330, 300.0243	Quercetin-7-glucoside- rhamnose
27	8.43	367.1025	0.2	C_17_H_20_O_9_	All	191.0562, 173.0446, 1111.0441	Methyl- cryptochlorogenic acid
28 *^a^*	8.69	417.1412	3.5	C_18_H_26_O_11_	All	237.0762, 191.0606	Methyl-secoxyloganin
29	9.43	463.0872	−2.1	C_21_H_20_O_12_	All	301.0365, 300.0290, 151.0034	Quercetin-7-glucoside
30	9.68	463.0868	−3.2	C_21_H_20_O_12_	All	301.0365, 300.0290	Hyperoside
31	9.69	593.1509	−0.1	C_27_H_30_O_14_	All	430.0789, 285.0349, 284.0281	Luteolin-7-glucoside-4′- rhamnose
32	9.92	447.0961	7.3	C_21_H_20_O_11_	All	285.0388	Luteolin-7-glucoside
33	10.10	609.1467	1.6	C_27_H_30_O_16_	All	315.0522, 314.0450	Isorhamnetin- 3-rutinoside
34	10.11	593.1505	−0.8	C_27_H_30_O_14_	All	285.0401, 284.0312	Luteolin-7-rutinoside
35 *^c^*	10.61	515.1187	−0.5	C_25_H_24_O_12_	All	353.0885, 335.0793, 191.0561, 179.0356, 173.0458, 135.0457	Isochlorogenic acid B
36 *^c^*	10.98	515.1192	0.3	C_25_H_24_O_12_	All	353.0870, 335.0770, 191.0547, 179.0329, 173.0436, 135.0428	Isochlorogenic acid A
37	11.02	447.0967	8.2	C_21_H_20_O_11_	All	285.0418, 284.0349	Kaempferol-3-glucoside
38	11.42	477.1078	9.3	C_22_H_22_O_12_	All	314.0441, 271.0264	Isorhamnetin-7-glucoside
39	11.60	431.1021	9.5	C_25_H_24_O_12_	All	269.0430, 268.0359	Apigenin-7-glucoside
40	11.63	193.0518	8.8	C_10_H_10_O_4_	All	161.0258, 134.0357, 133.0287	Isoferulic acid
41	11.73	193.0493	−4.1	C_10_H_10_O_4_	All	161.0243, 134.0384	Ferulic acid
42 *^c^*	12.05	515.1188	−0.3	C_25_H_24_O_12_	All	353.0886, 335.0749, 191.0554, 179.0353, 173.0460, 135.0462	Isochlorogenic acid C
43	12.42	537.1596	−1.1	C_25_H_30_O_13_	All	375.1291, 179.0343, 161.0254	Grandiforoside
44 ^*a*^	12.80	499.1287	10.0	C_25_H_24_O_11_	All	353.0931, 337.0893, 191.0558 179.0405	Dehydrogen- isochlorogenic acid A
45	12.89	491.1249	9.6	C_23_H_24_O_12_	All	329.0670	Tricin-7-glucoside
46	13.30	529.1350	−1.7	C_25_H_24_O_11_	All	367.1037, 353.0879, 191.0555, 179.0363, 161.0221	Methyl- isochlorogenic acid B
47	13.81	337.0930	2.0	C_16_H_18_O_9_	All	191.0560, 173.0453, 163.0400	3-*O*-p-coumaroylquinic acid
48 ^*a*^	13.90	499.1202	−7.0	C_25_H_24_O_11_	All	353.0844, 191.0575, 179.0396	Dehydrogen- isochlorogenic acid B
49 ^*a*^	14.24	529.1352	−1.3	C_25_H_24_O_11_	All	367.1027, 353.0862, 191.0549, 179.0337, 173.0435	Methyl- isochlorogenic acid A
50	14.60	367.1030	1.6	C_17_H_20_O_9_	All	193.0499, 129.0555, 101.0612	Methyl 4-caffeoylquinate
51	15.22	529.1329	−5.6	C_25_H_24_O_11_	All	367.1043, 179.0348, 161.0235	Methyl- isochlorogenic acid C
52	15.98	677.1505	−0.59	C_34_H_30_O_15_	All	515.1188, 353.0865, 173.0418	3,4,5-tricaffeoylquinic acid
53 *^a^*	17.15	1397.6613	1.7	C_65_H_106_O_32_	All	1073.5528, 643.1071	Dipsacoside B- diglucoside
54 ^*a*^	17.77	1559.7124	0.4	C_71_H_116_O_37_	All	1235.6122, 1073.5249, 652.6374	Dipsacoside B- triglucoside
55 ^*a*^	17.82	1543.7128	−2.5	C_71_H_116_O_36_	All	1381.6549, 1219.6052	Dipsacoside B-rutinoside-glucoside
56 ^*a*,*b*^	18.01	1135.5944	4.7	C_53_H_86_O_23_	All	1089.5447, 765.4313, 323.0779	Hydroxyl-dipsacoside B
57	18.25	1397.6597	0.4	C_65_H_106_O_32_	All	1073.5499	Isodipsacoside B- diglucoside
58 ^*a*^	18.54	1235.6060	4.7	C_59_H_96_O_27_	All	1073.5509, 911.4954, 527.3396	Dipsacoside B-glucoside
59	18.70	1235.6084	5.8	C_59_H_96_O_27_	All	911.5006, 749.4477	Isodipsacoside-B- glucoside
60 ^*a*^	18.98	327.2184	3.6	C_18_H_32_O_5_	All	229.1477, 221.1116, 211.1333	Dehydroxypinellic acid
61 *^a^*^,*b*^	19.00	1105.5577	2.1	C_52_H_84_O_22_	All	1059.5310, 889.1119, 735.4228, 469.7788, 323.0942	Demethyl-dipsacoside B
62	19.03	329.0658	0.6	C_17_H_14_O_7_	All	167.0344, 152.0089	Tricin
63 ^*b*^	19.16	1119.5466	0.3	C_53_H_86_O_22_	All	1073.5531, 749.4500, 323.0951	Dipsacoside B
64 ^*b*^	19.25	1119.5545	8.7	C_53_H_86_O_22_	All	1073.5517, 911.5158, 749.4457, 323.0971	Macranthoside B
65 ^*a*^	19.30	1395.6466	2.3	C_65_H_104_O_32_	All	1071.5376	Dehydrogen- dipsacoside B-maltose
66	19.38	1119.5559	9.3	C_53_H_86_O_22_	All	1073.5501, 911.5094, 749.4431, 323.1027	Isodipsacoside B
67 ^*a*^	20.55	1247.7241	5.6	C_65_H_106_O_32_	All	1381.6554, 1238.1004, 1057.5537	Dipsacoside B-rutinoside
68 ^*a*^	20.73	329.2340	3.6	C_18_H_34_O_5_	All	229.1538, 171.1070	Pinellic acid
69 ^*a*^	20.82	1381.6681	2.9	C_65_H_106_O_32_	All	1057.5602	Dipsacoside B- glucoside-rhamnose
70 ^*a*^	21.23	1219.6150	3.1	C_59_H_96_O_26_	All	895.5042, 733.4157, 517.0624	Dipsacoside B-rhamnose
71 ^*a*^	22.13	1103.5547	−3.4	C_52_H_82_O_22_	All	1057.5556, 733.4597, 323.0945	Demethyl-dehydrogen- dipsacoside B
72 ^*a*^	25.73	911.4995	−1.0	C_47_H_76_O_17_	All	749.4421, 603.3780	Deglucoside- dipsacoside B

*^a^* indicates the compound was reported for the first time from *L. japonica* and *L. hypoglauca*; *^b^* indicates the [M − H + HCOO]−; *^c^* indicates the compound was unambiguously determined by comparing the *t*_R_, MS, and MS/MS with the references.

## Data Availability

Data are contained within the article and Supplementary Materials.

## Supplementary Materials

The following supporting information can be downloaded at: https://www.mdpi.com/article/10.3390/molecules29204848/s1, Figure S1: The MS/MS spectra of compounds **1**–**72**; Table S1: The components of high-fat diet. Table S2: The groups, number of mice, and dose design of hypoglycemic experiment for *L. japonica* buds, *L. hypoglauca* buds, roots, stems, and leaves extracts; Table S3: The groups, number of mice, and dose design of hypoglycemic experiment for seven compounds.
