# Supplementary material for: Identification of the Hypoglycemic Active Components of Lonicera japonica Thunb. and Lonicera hypoglauca Miq. by UPLC-Q-TOF-MS
Source: Molecules. 2024 Oct 13;29(20):4848. doi: 10.3390/molecules29204848 (PMC11510595; doi:10.3390/molecules29204848)
Supplement: Supplementary file 1 [file molecules-29-04848-s001.zip › molecules-3231469-supplementary.pdf]

molecules

MDPI

Supplementary Materials

Identification of the hypoglycemic active components of *Lonicera japonica* Thunb. and *Lonicera hypoglauca* Miq. by UPLC-Q-TOF-MS

Qinxuan Wu[1], Di Zhao[1], Ying Leng[2], Canhui Chen[1], Kunyu Xiao[1], Zhaoquan Wu[1], Fengming Chen[1*]

[1] Hunan Provincial Key Laboratory of the Traditional Chinese Medicine Agricultural Biogenomics, The "Double-First Class" Application Characteristic Discipline of Hunan Province (Pharmaceutical Science), Changsha Medical University, Changsha 410219, China; qinxuanwucsmu@163.com (Q.W.); didixiao0806@163.com (D.Z.); csyxy613@163.com (C.C); 932071172@qq.com (K.X.); 402047686@qq.com (Z.W.); cfming@csmu.edu.cn (F.C.)

[2] Hunan Pharmaceutical Development and investment Group Co. Ltd:, Changsha, 410219, China; 582962392@qq.com (Y.L.)

* Correspondence: cfming@csmu.edu.cn (F.C.); Tel.: +86-731-84377621.

Fig. S1 the MS/MS spectra of compounds **1-60**.

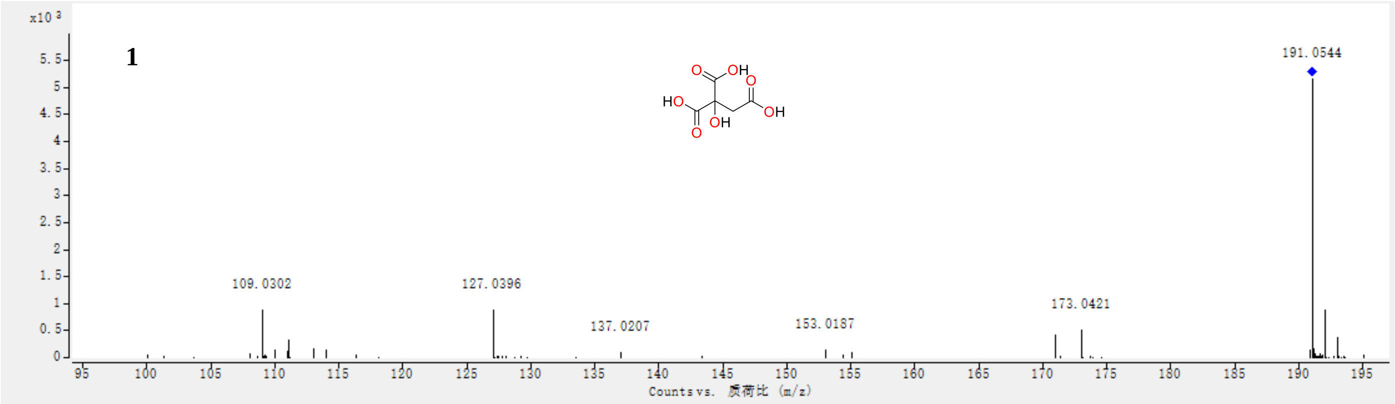

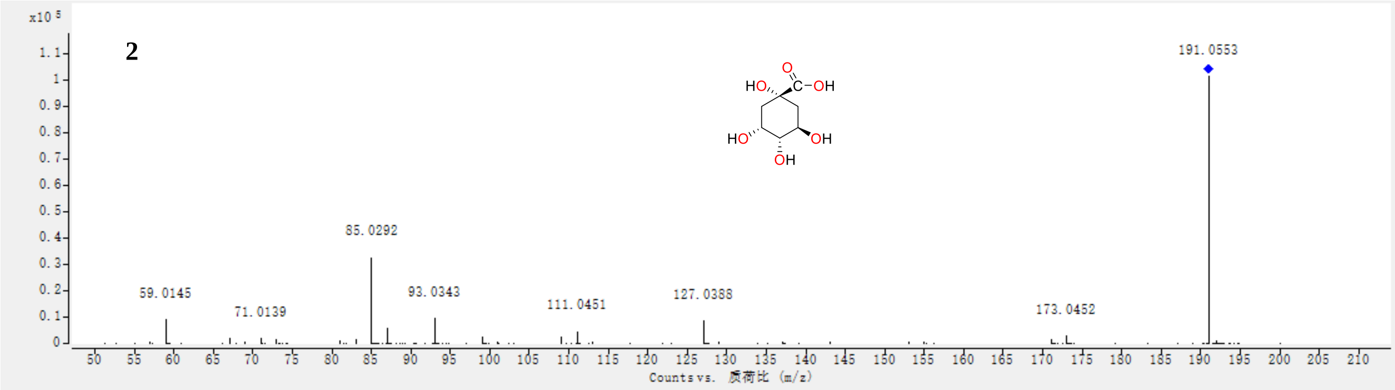

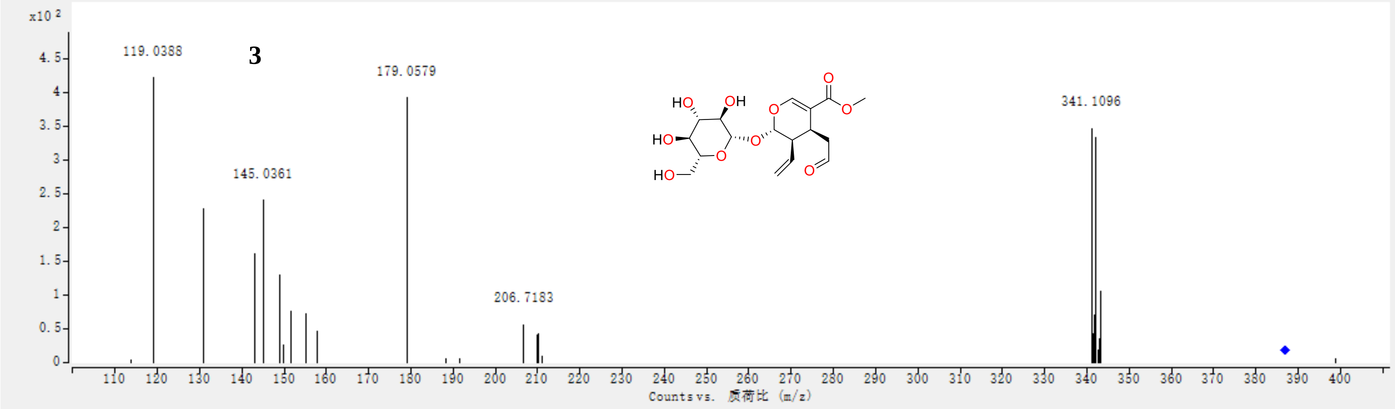

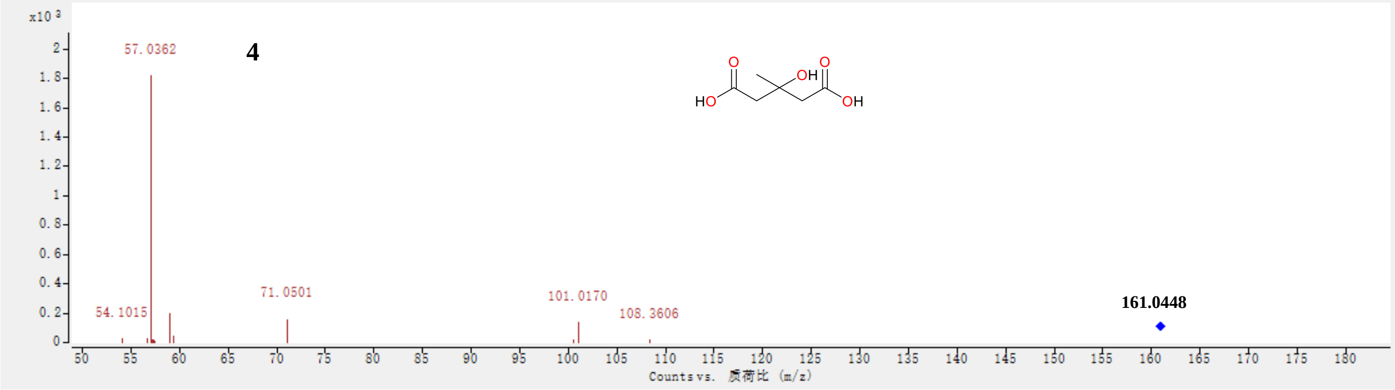

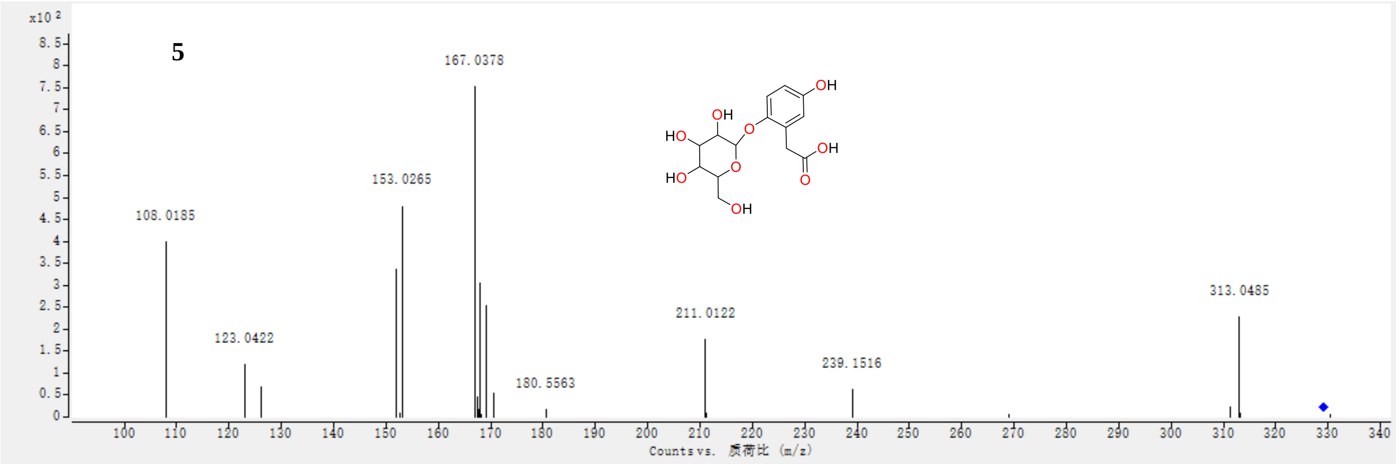
5
x10 2
108. 0185
123. 0422
153. 0265
167. 0378
180. 5563
211. 0122
239. 1516
313. 0485
Counts vs. 质荷比 (m/z)

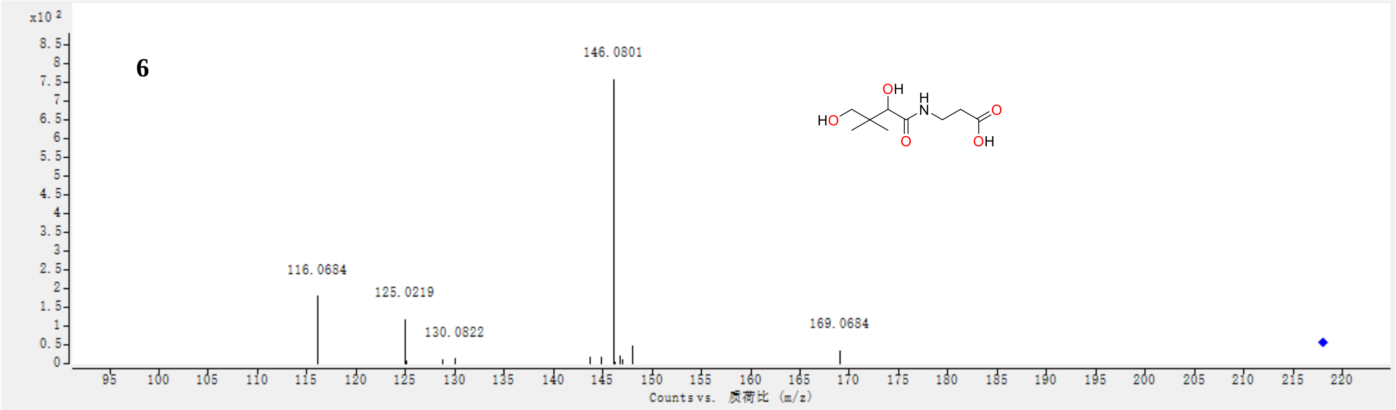
6
x10 2
116. 0684
125. 0219
130. 0822
146. 0801
169. 0684
Counts vs. 质荷比 (m/z)

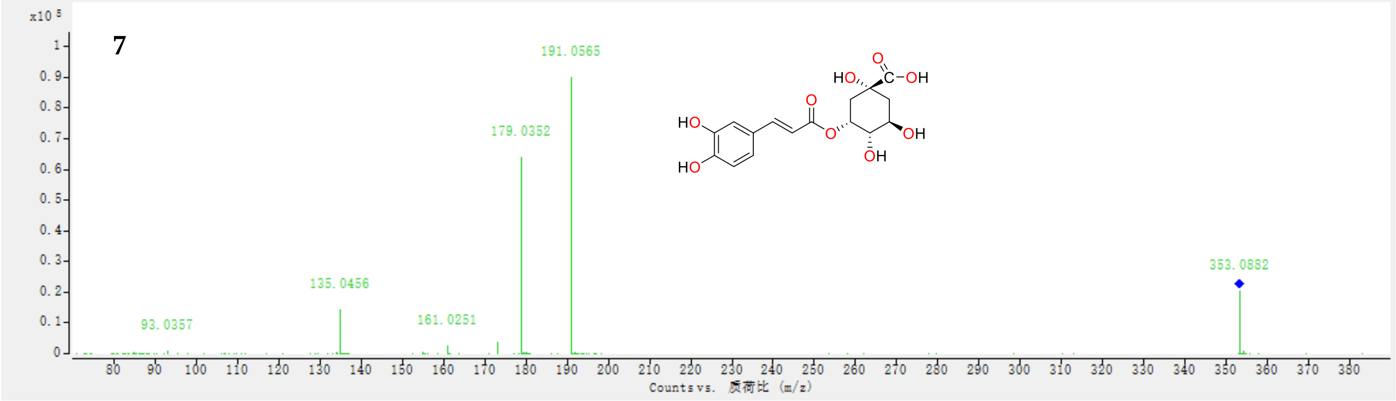
7
x10 5
93. 0357
135. 0456
161. 0251
179. 0352
191. 0565
353. 0882
Counts vs. 质荷比 (m/z)

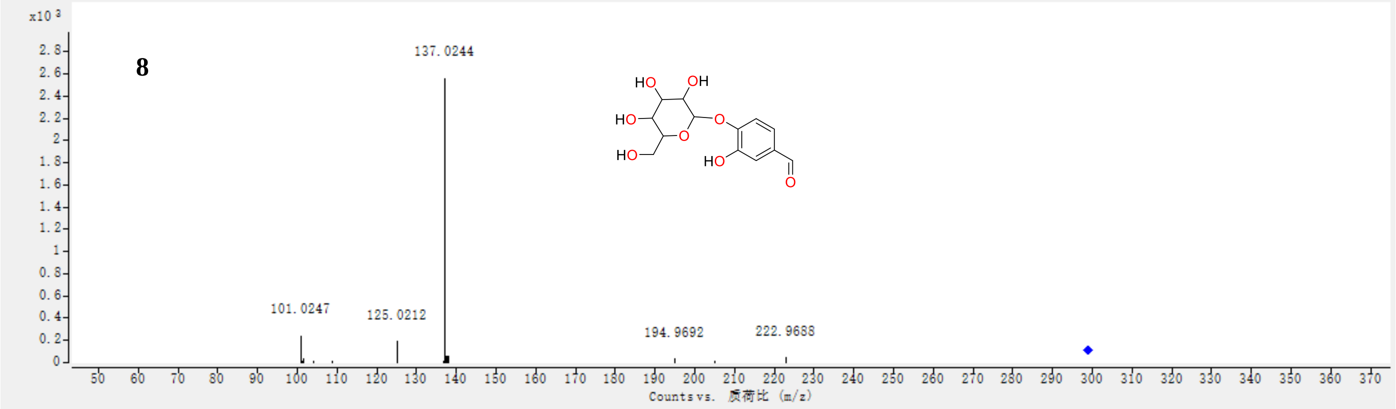
8
x10 3
101. 0247
125. 0212
137. 0244
194. 9692
222. 9688
Counts vs. 质荷比 (m/z)

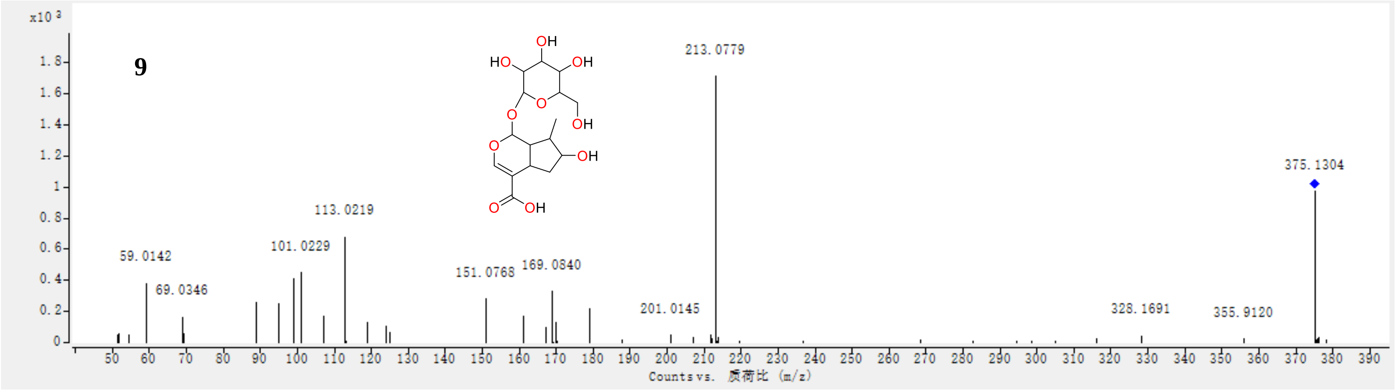
9
213. 0779
375. 1304
113. 0219
59. 0142
101. 0229
69. 0346
151. 0768
169. 0840
201. 0145
328. 1691
355. 9120
Counts vs. 质荷比 (m/z)

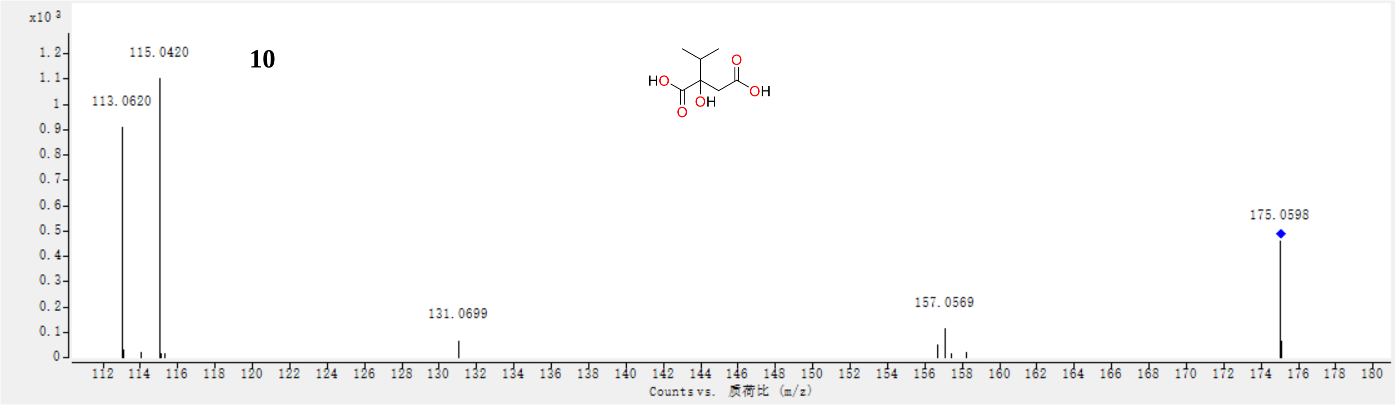
10
115. 0420
113. 0620
131. 0699
157. 0569
175. 0598
Counts vs. 质荷比 (m/z)

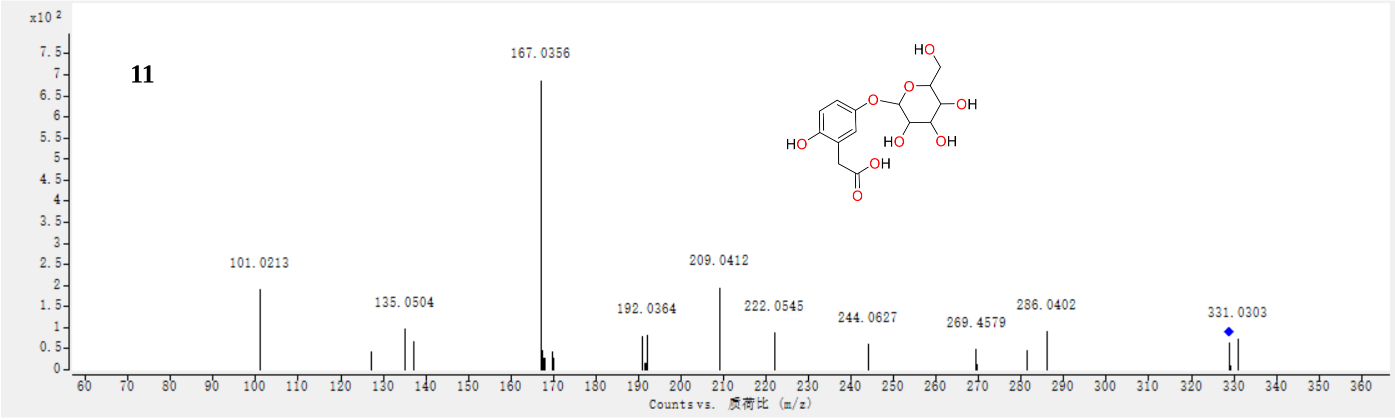
11
167. 0356
101. 0213
135. 0504
192. 0364
209. 0412
222. 0545
244. 0627
269. 4579
286. 0402
331. 0303
Counts vs. 质荷比 (m/z)

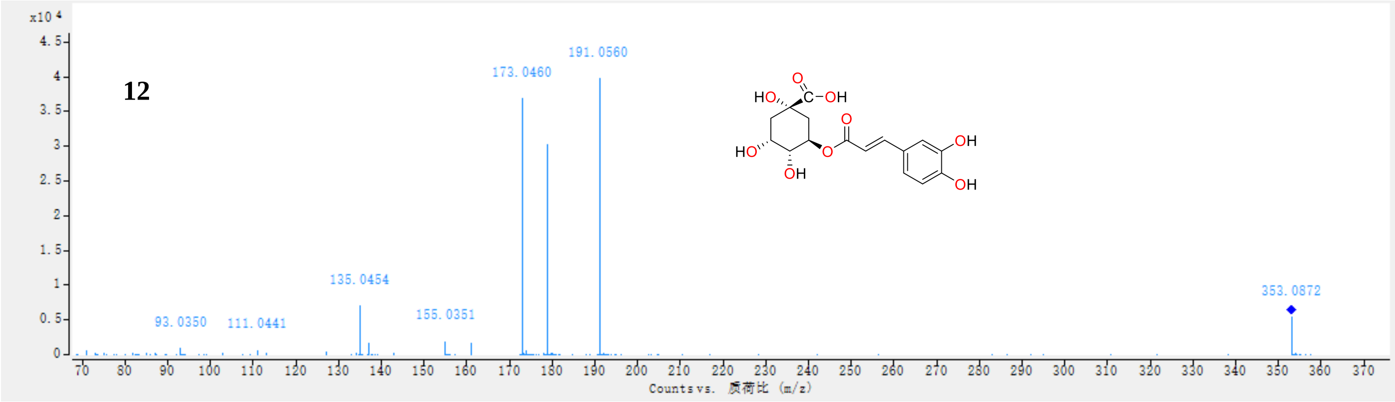
12
191. 0560
173. 0460
135. 0454
93. 0350
111. 0441
155. 0351
353. 0872
Counts vs. 质荷比 (m/z)

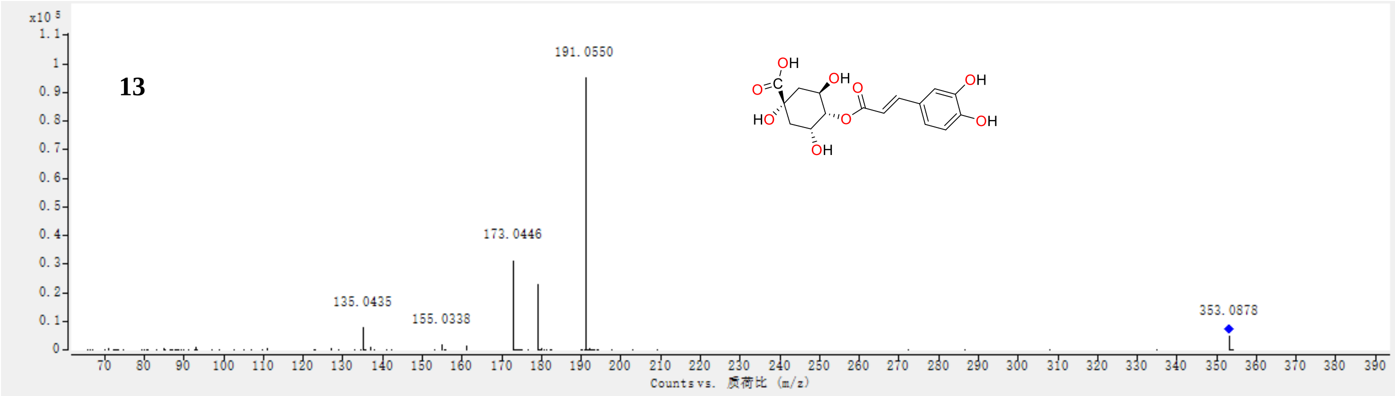
x10 5
13
191. 0550
173. 0446
135. 0435
155. 0338
353. 0878
Counts vs. 质荷比 (m/z)

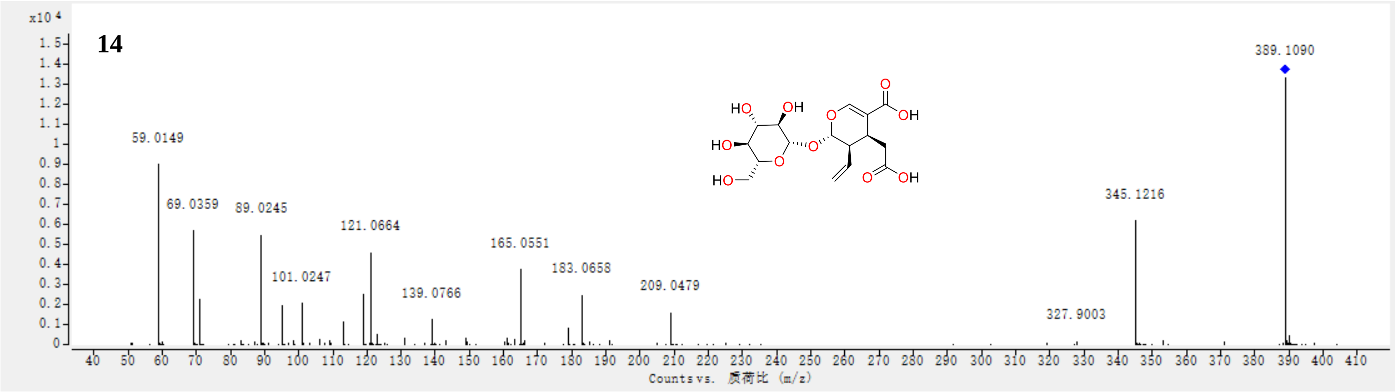
x10 4
14
389. 1090
59. 0149
69. 0359
89. 0245
121. 0664
101. 0247
139. 0766
165. 0551
183. 0658
209. 0479
327. 9003
345. 1216
Counts vs. 质荷比 (m/z)

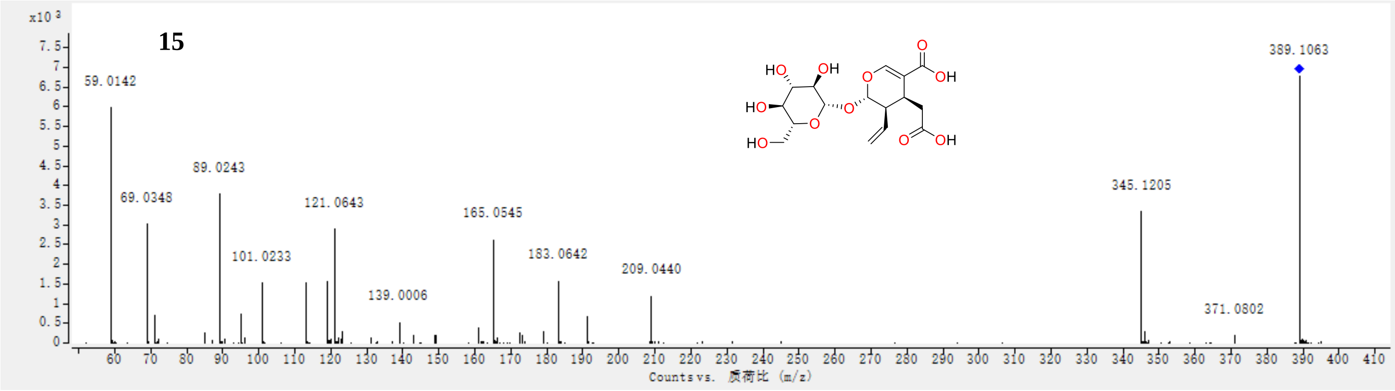
x10 3
15
59. 0142
69. 0348
89. 0243
101. 0233
121. 0643
139. 0006
165. 0545
183. 0642
209. 0440
345. 1205
371. 0802
389. 1063
Counts vs. 质荷比 (m/z)

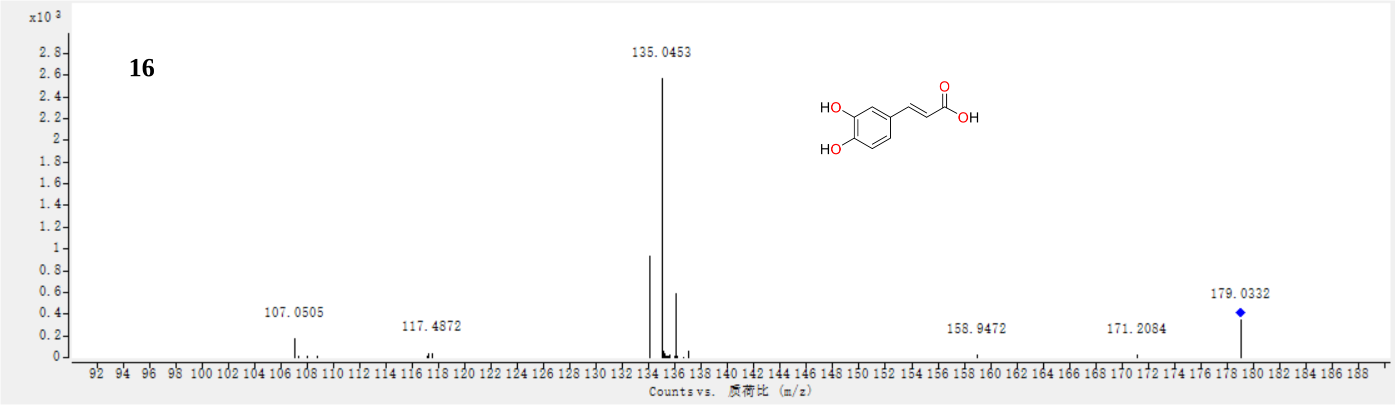
x10 3
16
135. 0453
107. 0505
117. 4872
158. 9472
171. 2084
179. 0332
Counts vs. 质荷比 (m/z)

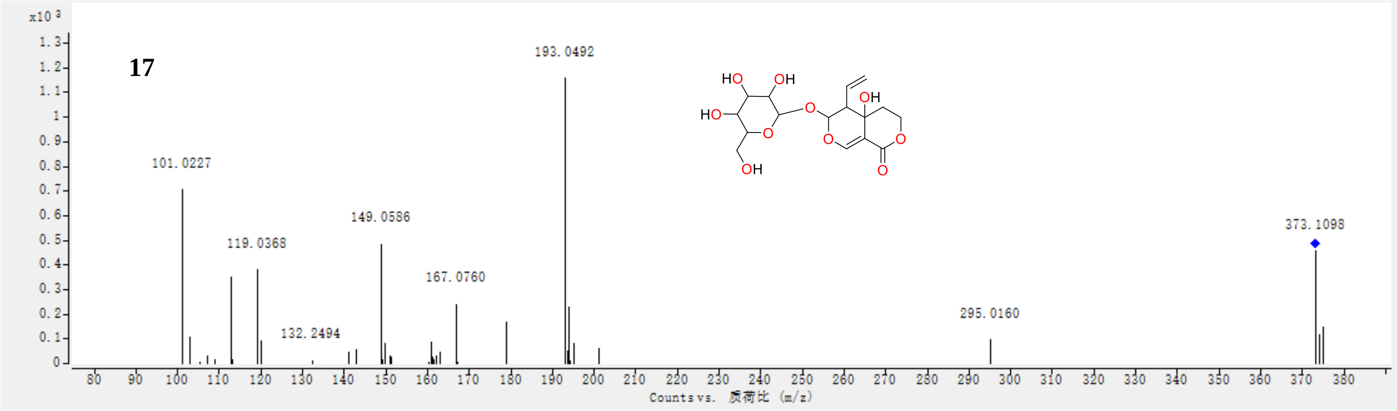
x10 3
17
193. 0492
101. 0227
119. 0368
132. 2494
149. 0586
167. 0760
295. 0160
373. 1098
Counts vs. 质荷比 (m/z)

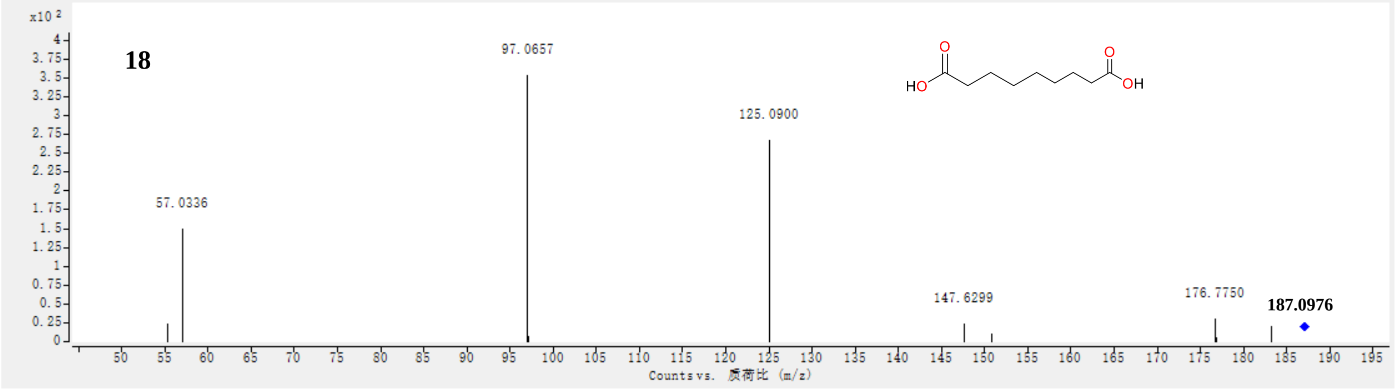
x10 2
18
97. 0657
125. 0900
57. 0336
147. 6299
176. 7750
187.0976
Counts vs. 质荷比 (m/z)

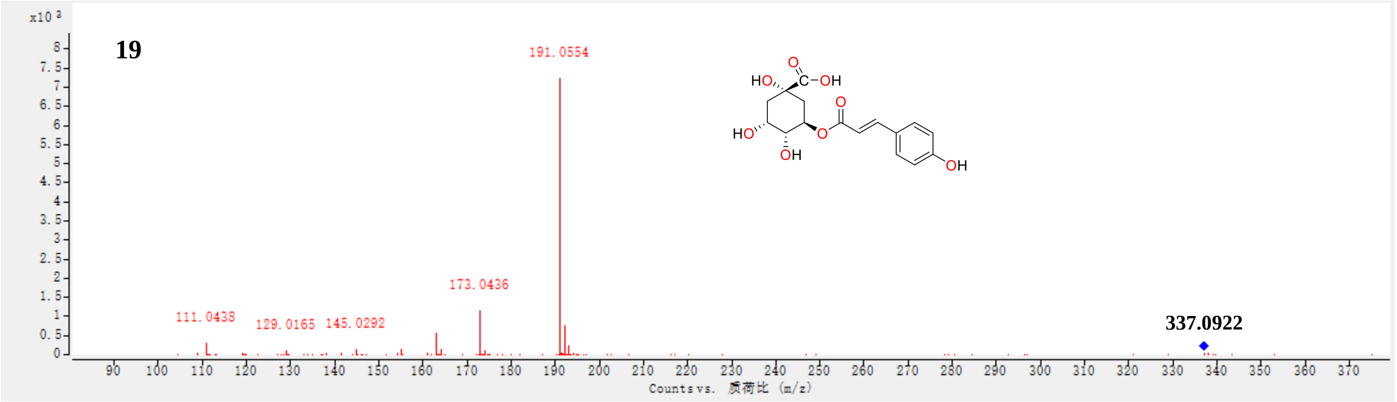
x10 3
19
191. 0554
173. 0436
111. 0438
129. 0165
145. 0292
337.0922
Counts vs. 质荷比 (m/z)

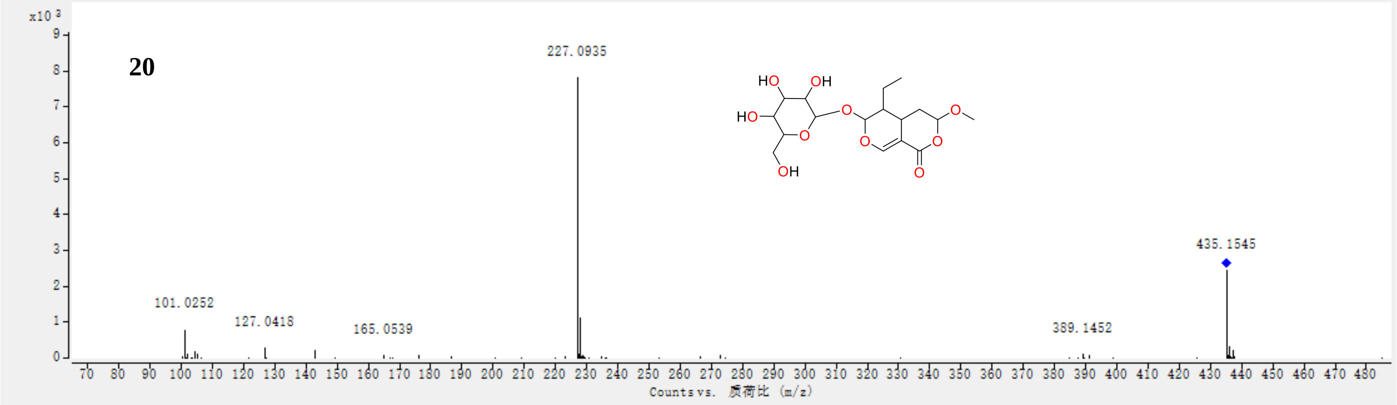
x10 3
20
227. 0935
101. 0252
127. 0418
165. 0539
389. 1452
435. 1545
Counts vs. 质荷比 (m/z)

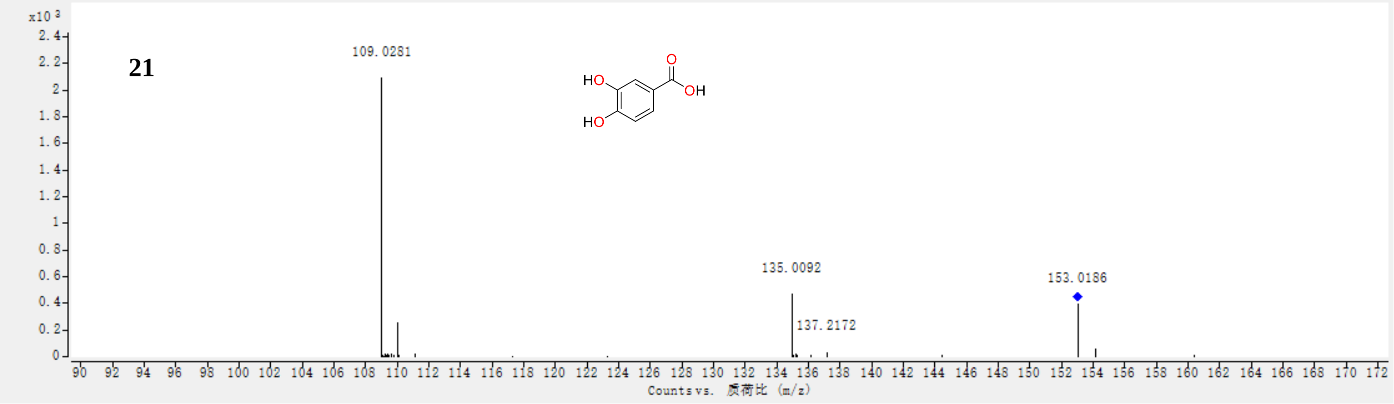
x10 3
21
109. 0281
135. 0092
137. 2172
153. 0186
HO
HO
O
OH
Counts vs. 质荷比 (m/z)

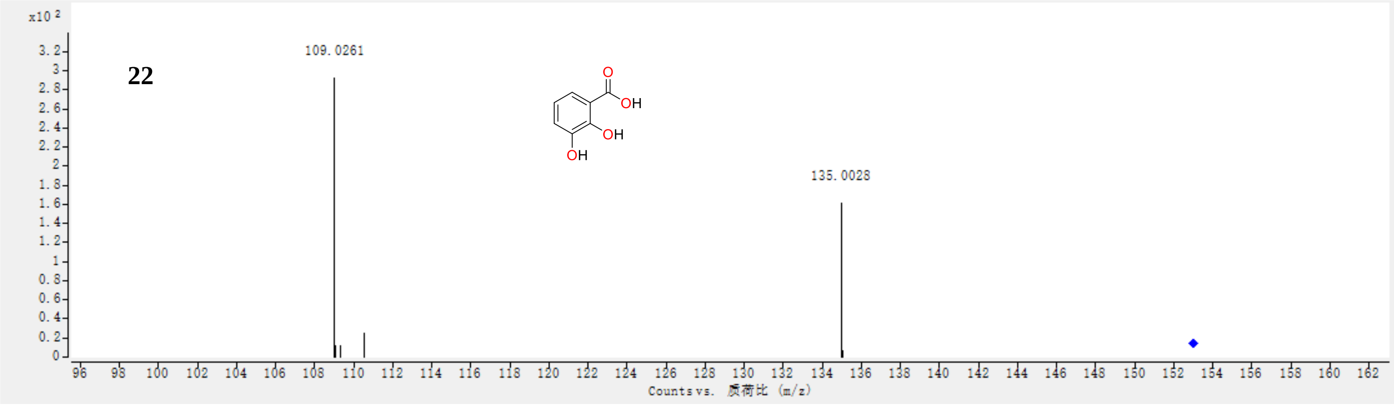
x10 2
22
109. 0261
135. 0028
O
OH
OH
OH
Counts vs. 质荷比 (m/z)

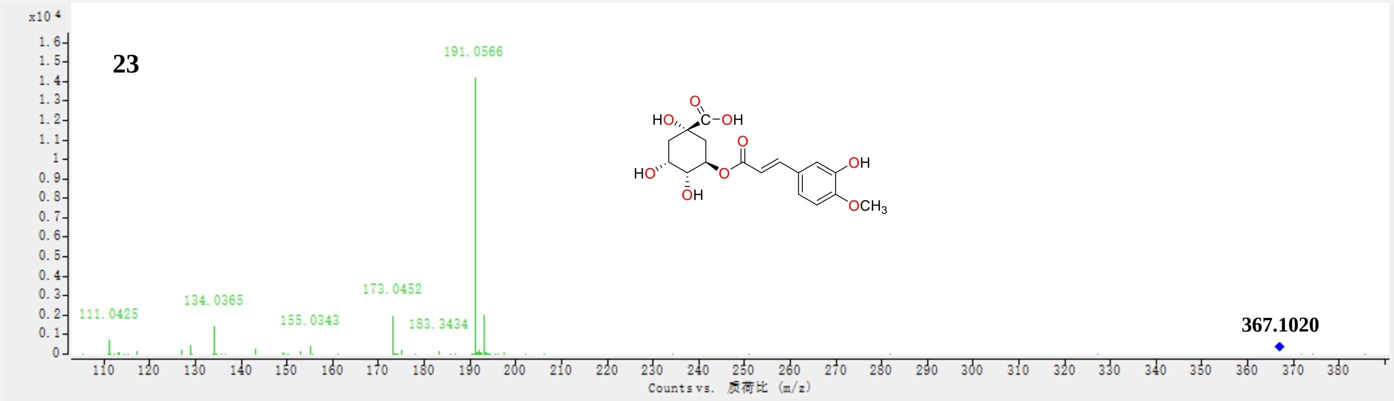
x10 4
23
191. 0566
111. 0425
134. 0365
155. 0343
173. 0452
183. 3434
367.1020
HO
C–OH
HO
OH
O
O
OH
OCH3
Counts vs. 质荷比 (m/z)

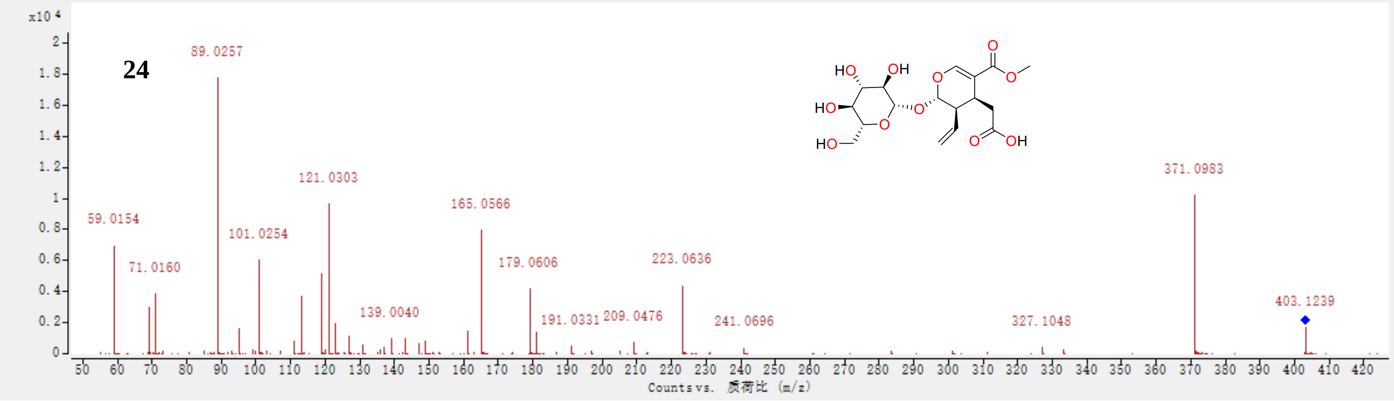
x10 4
24
89. 0257
59. 0154
71. 0160
101. 0254
121. 0303
139. 0040
165. 0566
179. 0606
191. 0331
209. 0476
223. 0636
241. 0696
327. 1048
371. 0983
403. 1239
HO
OH
HO
HO
O
O
O
O
O
OH
Counts vs. 质荷比 (m/z)

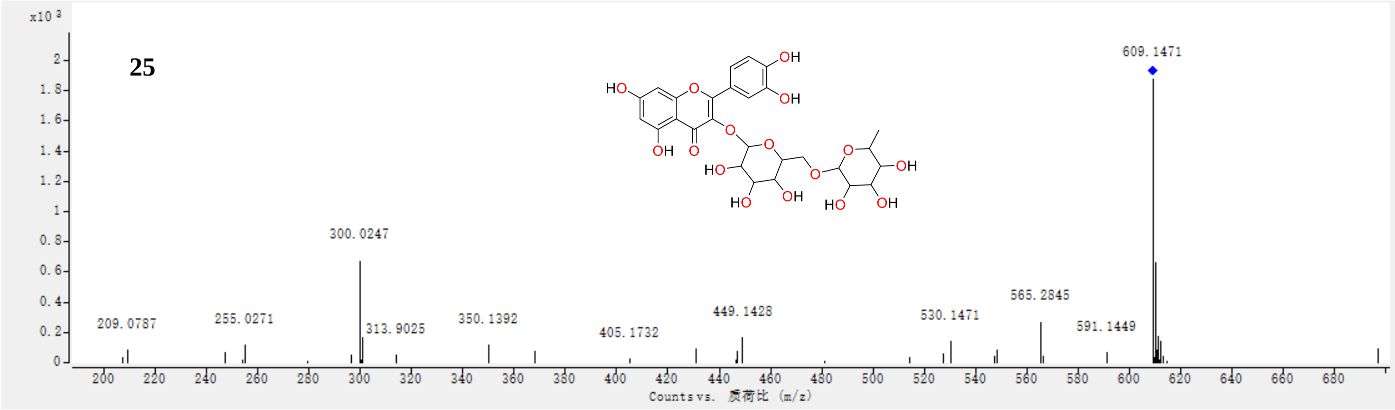
x10 3
25
609.1471
300.0247
209.0787
255.0271
313.9025
350.1392
405.1732
449.1428
530.1471
565.2845
591.1449
Counts vs. 质荷比 (m/z)

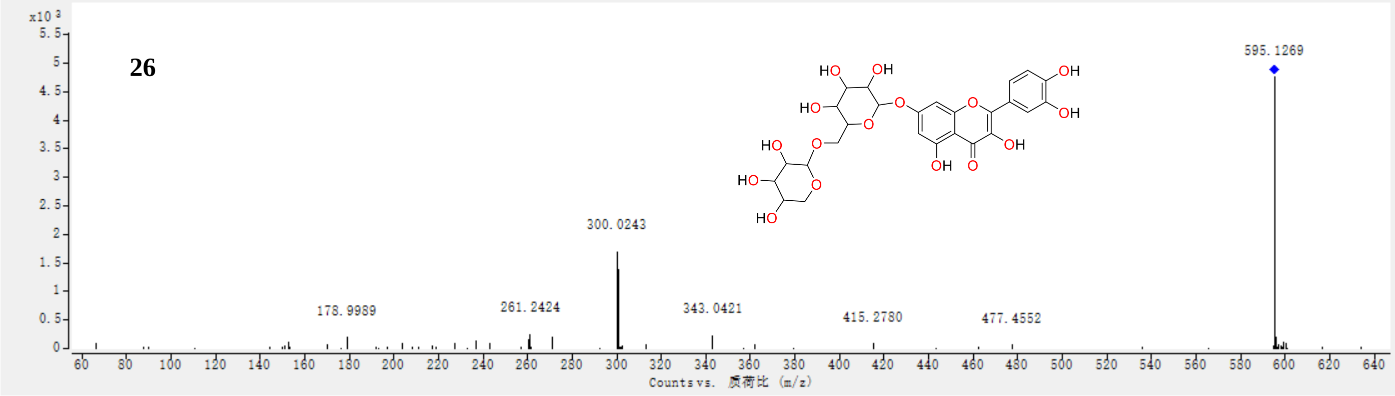
x10 3
26
595.1269
300.0243
178.9989
261.2424
343.0421
415.2780
477.4552
Counts vs. 质荷比 (m/z)

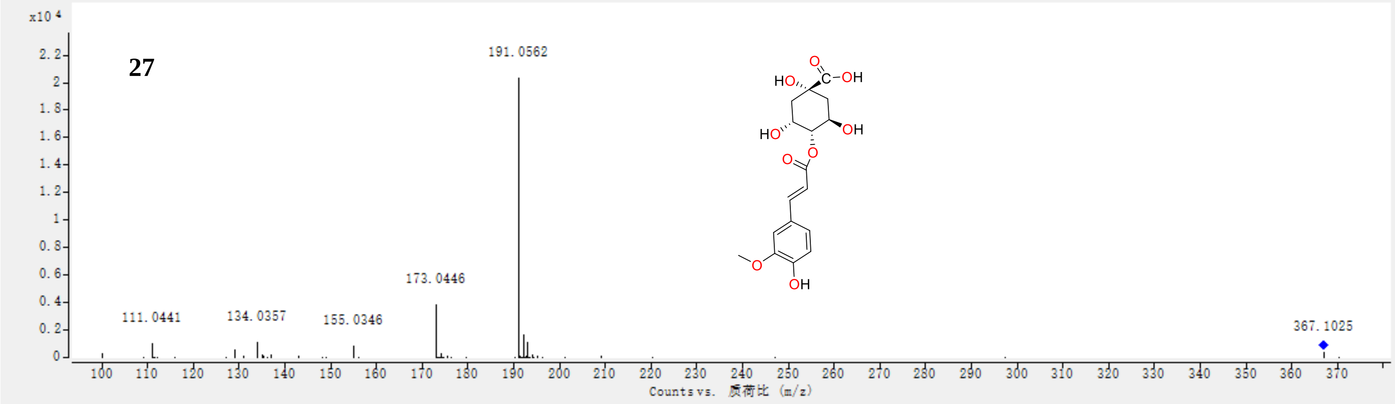
x10 4
27
191.0562
173.0446
111.0441
134.0357
155.0346
367.1025
Counts vs. 质荷比 (m/z)

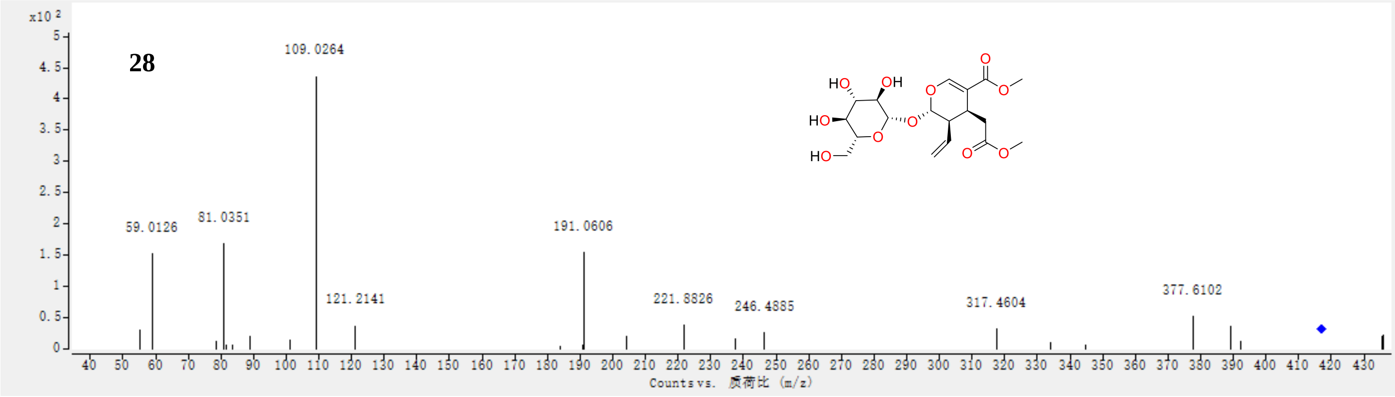
x10 2
28
109.0264
59.0126
81.0351
121.2141
191.0606
221.8826
246.4885
317.4604
377.6102
Counts vs. 质荷比 (m/z)

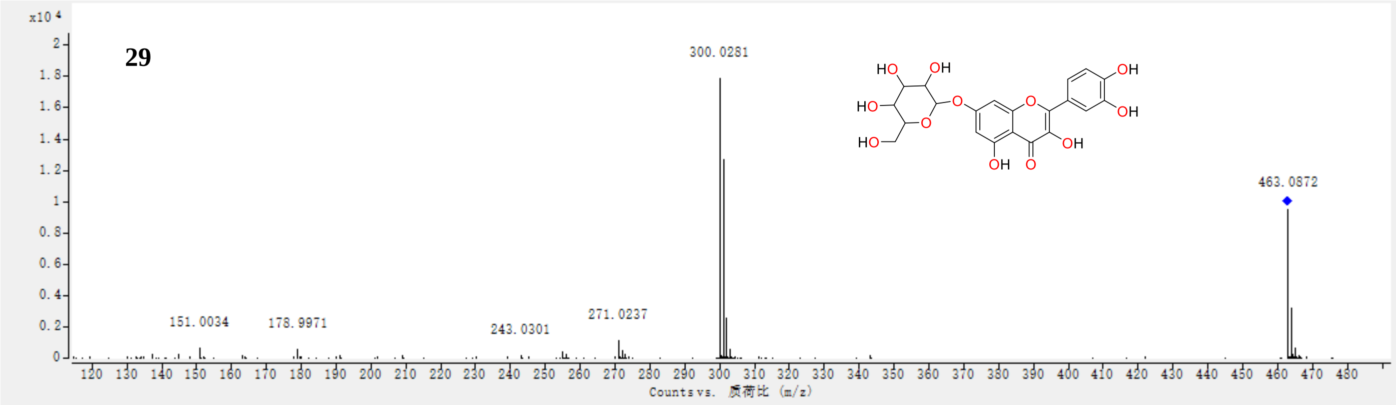
x10 4
29
300. 0281
463. 0872
151. 0034
178. 9971
243. 0301
271. 0237
Counts vs. 质荷比 (m/z)

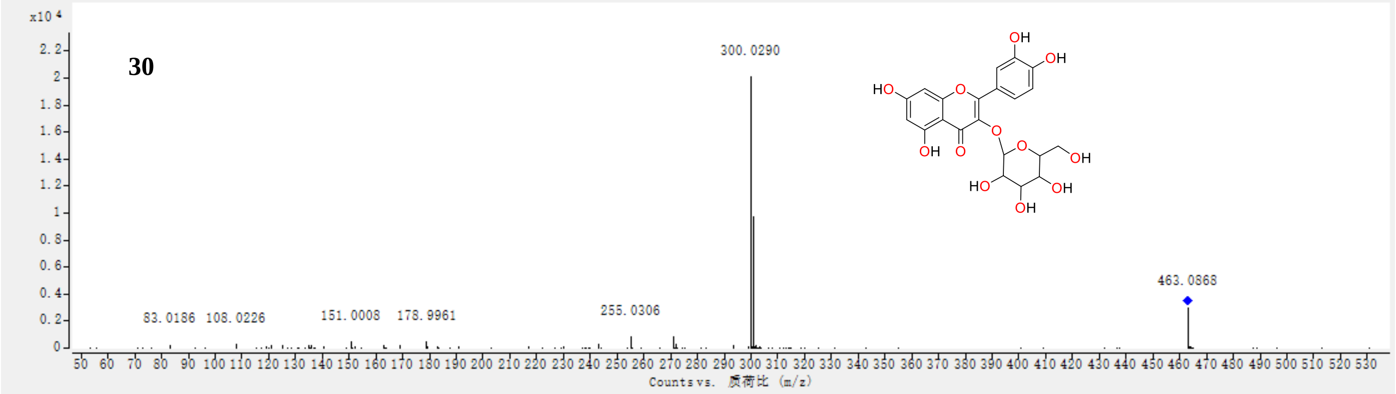
x10 4
30
300. 0290
463. 0868
83. 0186
108. 0226
151. 0008
178. 9961
255. 0306
Counts vs. 质荷比 (m/z)

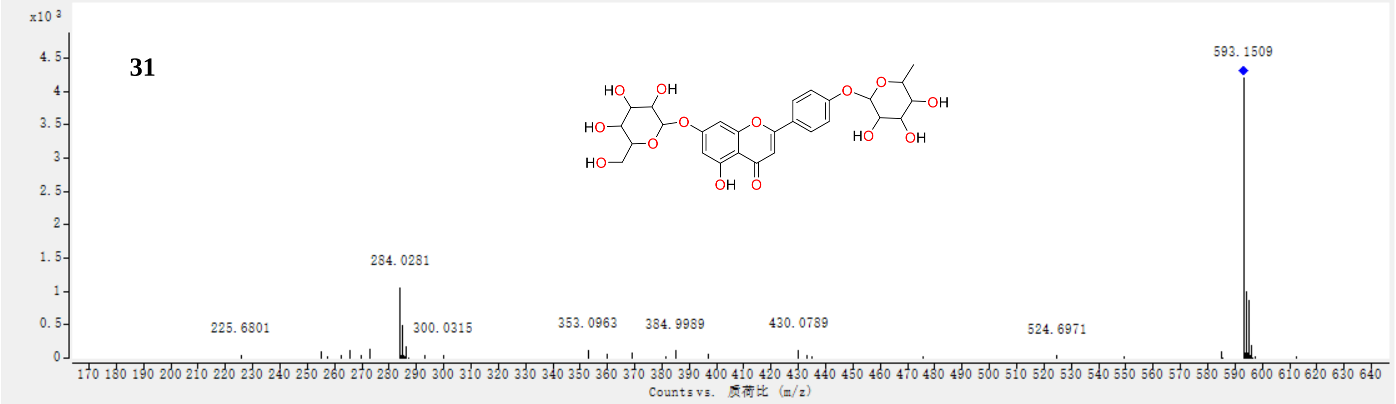
x10 3
31
593. 1509
284. 0281
225. 6801
300. 0315
353. 0963
384. 9989
430. 0789
524. 6971
Counts vs. 质荷比 (m/z)

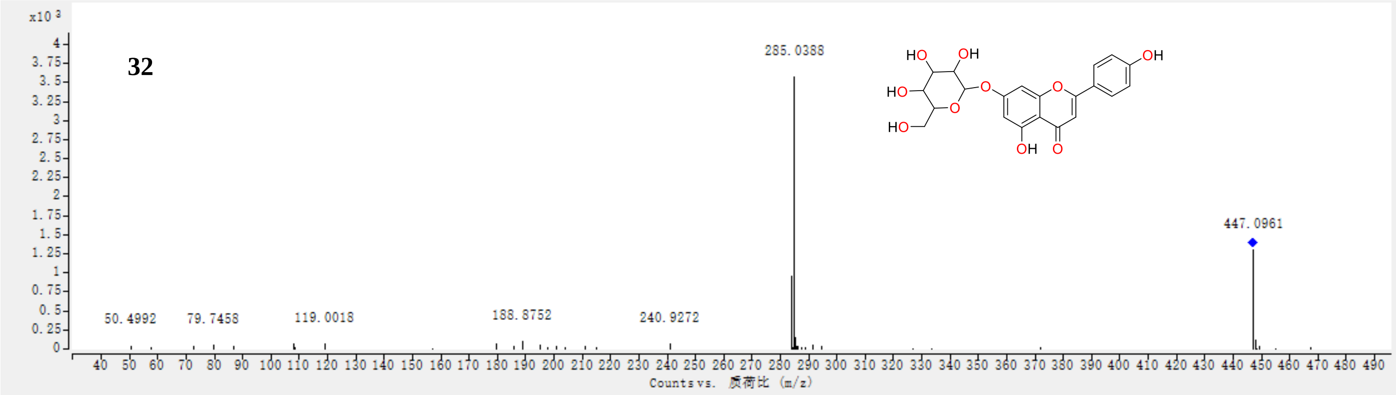
x10 3
32
285. 0388
447. 0961
50. 4992
79. 7458
119. 0018
188. 8752
240. 9272
Counts vs. 质荷比 (m/z)

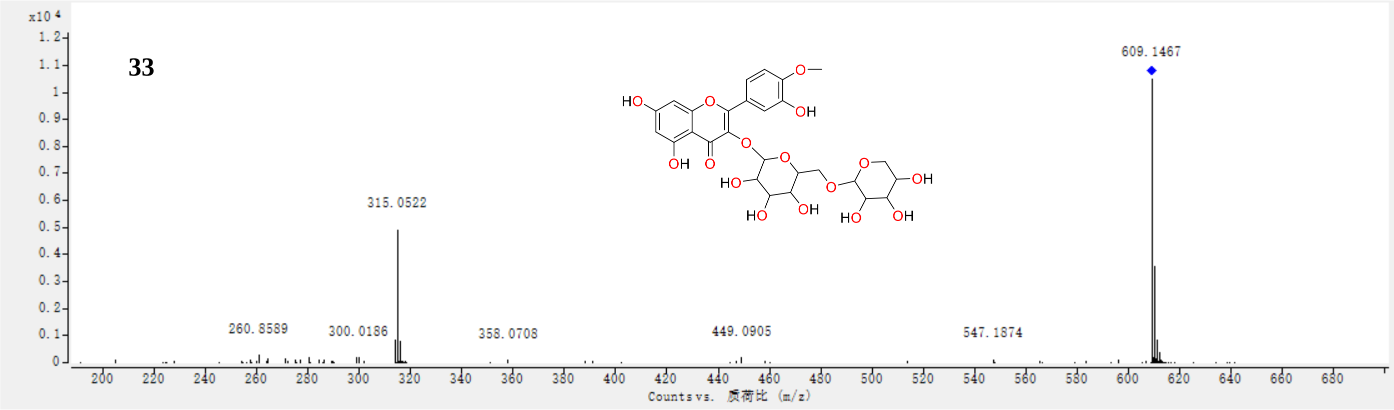
33
x10 4
609. 1467
315. 0522
260. 8589
300. 0186
358. 0708
449. 0905
547. 1874
Counts vs. 质荷比 (m/z)

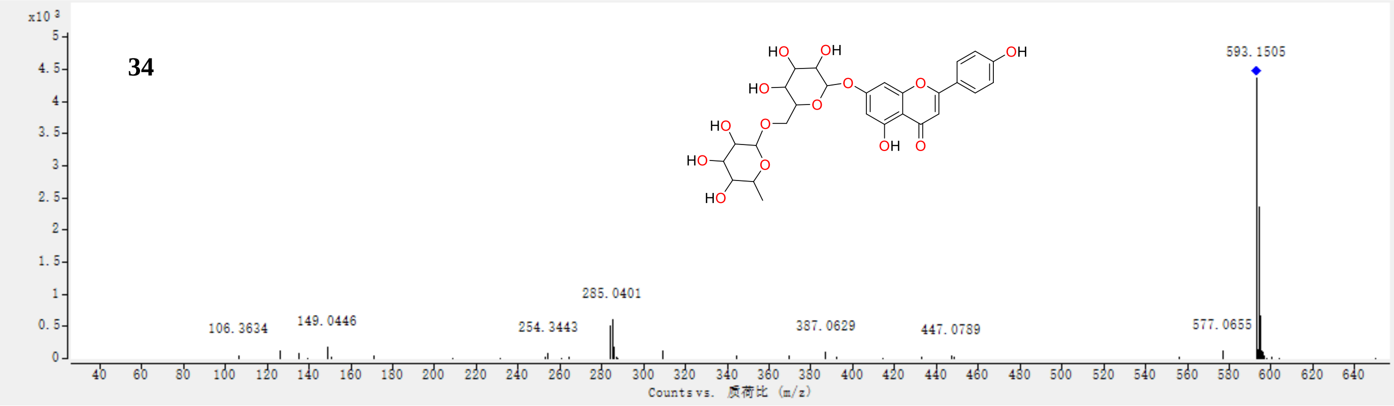
34
x10 3
593. 1505
285. 0401
106. 3634
149. 0446
254. 3443
387. 0629
447. 0789
577. 0655
Counts vs. 质荷比 (m/z)

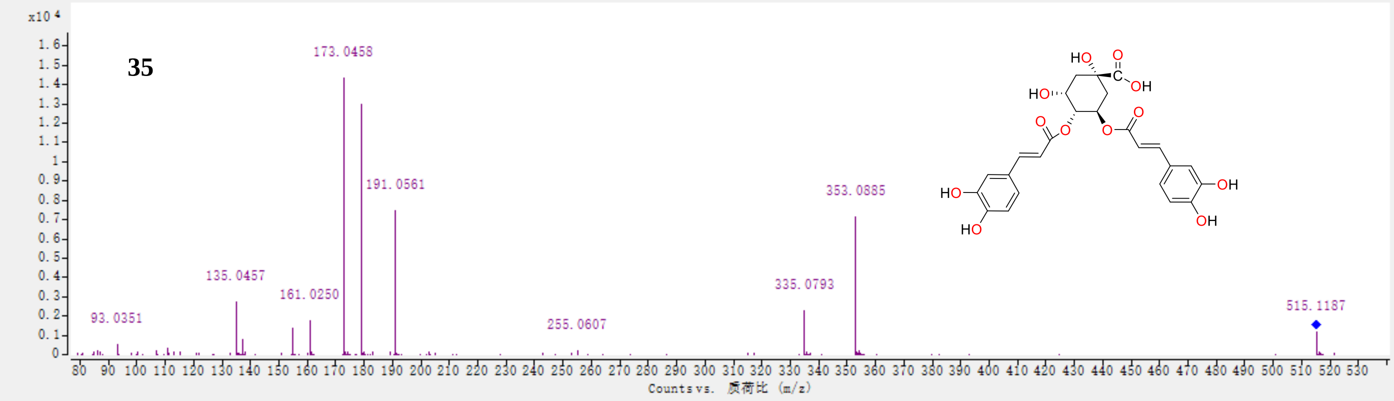
35
x10 4
173. 0458
191. 0561
353. 0885
135. 0457
161. 0250
93. 0351
255. 0607
335. 0793
515. 1187
Counts vs. 质荷比 (m/z)

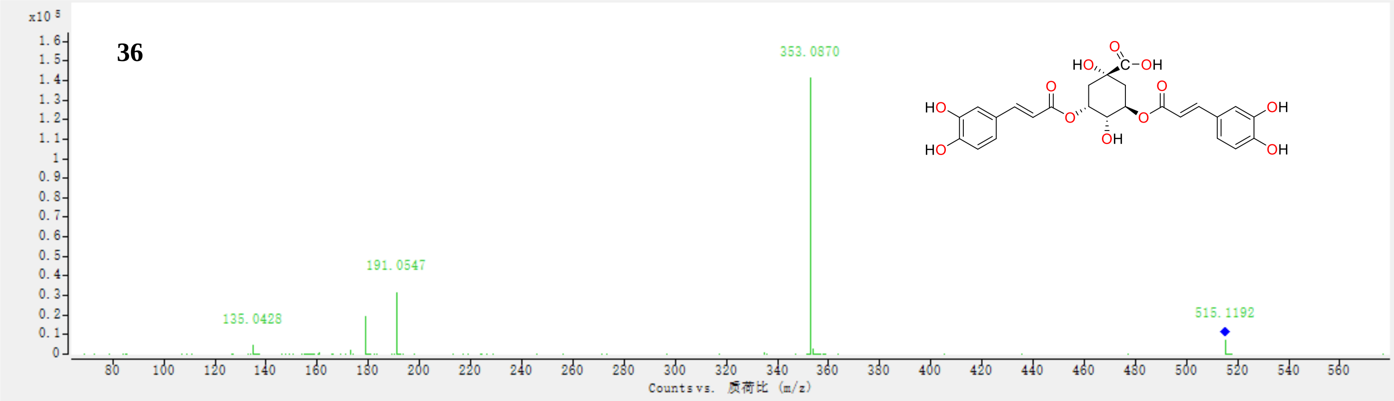
36
x10 5
353. 0870
191. 0547
135. 0428
515. 1192
Counts vs. 质荷比 (m/z)

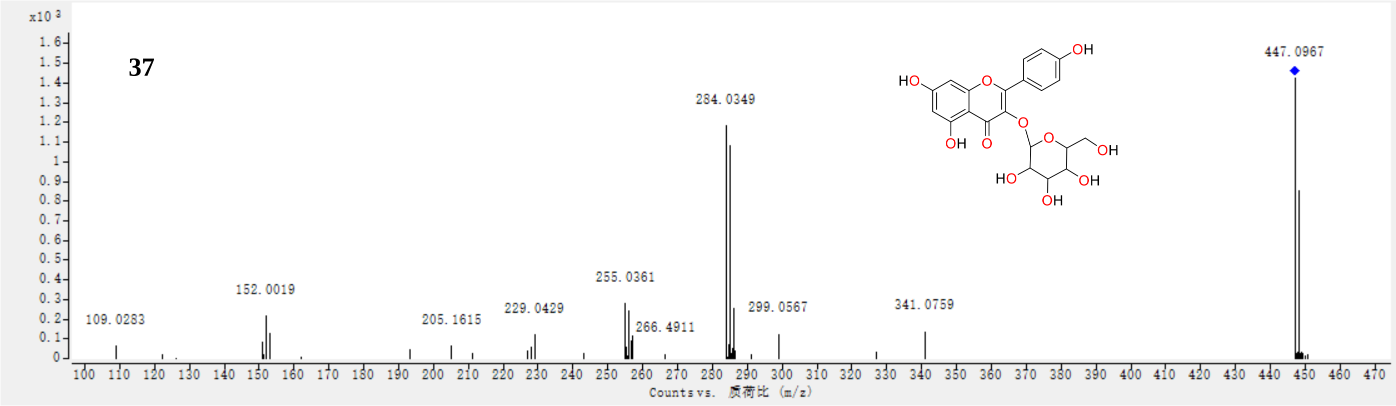
37
x10 3
447. 0967
284. 0349
152. 0019
109. 0283
205. 1615
229. 0429
255. 0361
266. 4911
299. 0567
341. 0759
Counts vs. 质荷比 (m/z)

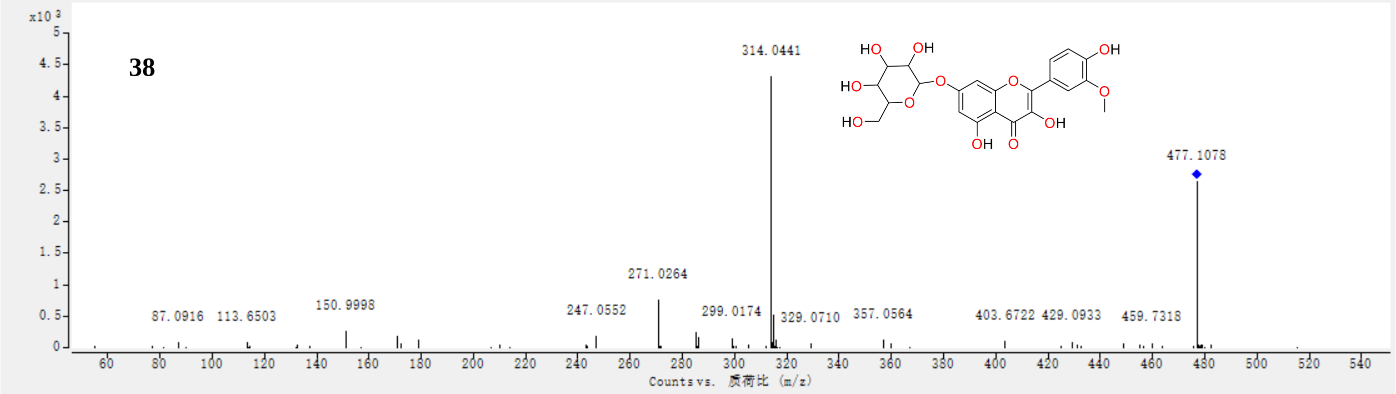
38
x10 3
314. 0441
477. 1078
271. 0264
87. 0916
113. 6503
150. 9998
247. 0552
299. 0174
329. 0710
357. 0564
403. 6722
429. 0933
459. 7318
Counts vs. 质荷比 (m/z)

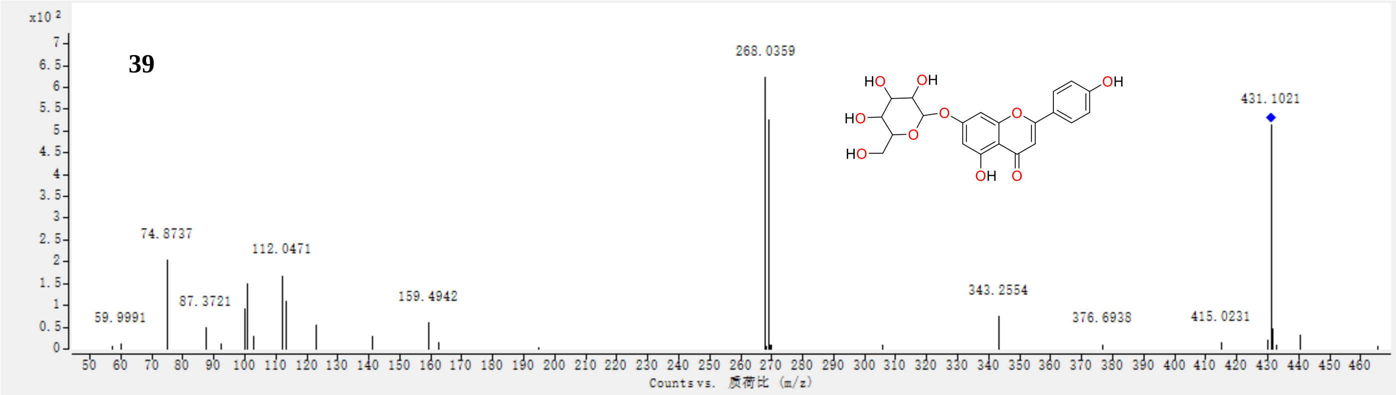
39
x10 2
268. 0359
431. 1021
74. 8737
112. 0471
87. 3721
59. 9991
159. 4942
343. 2554
376. 6938
415. 0231
Counts vs. 质荷比 (m/z)

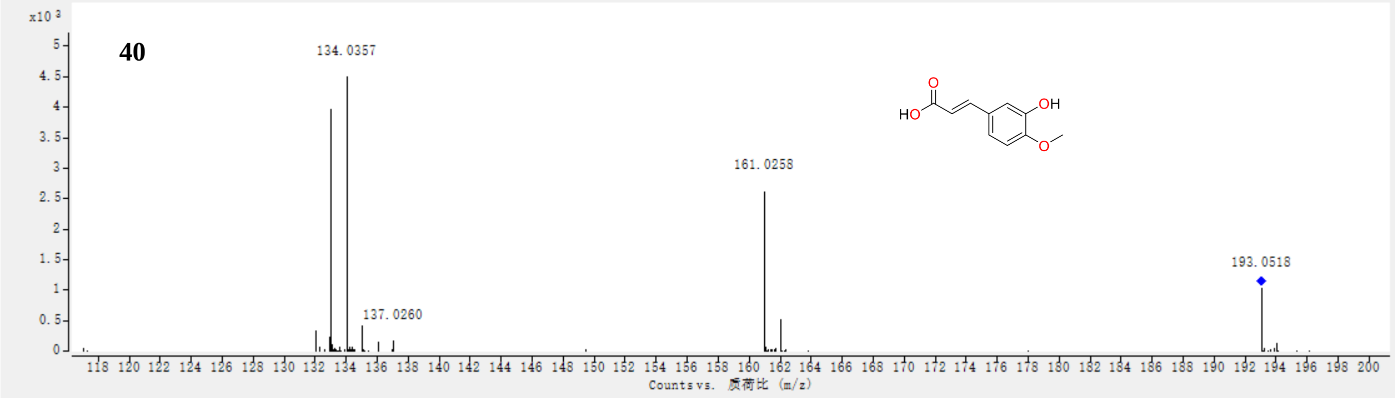
40
x10 3
134. 0357
161. 0258
193. 0518
137. 0260
Counts vs. 质荷比 (m/z)

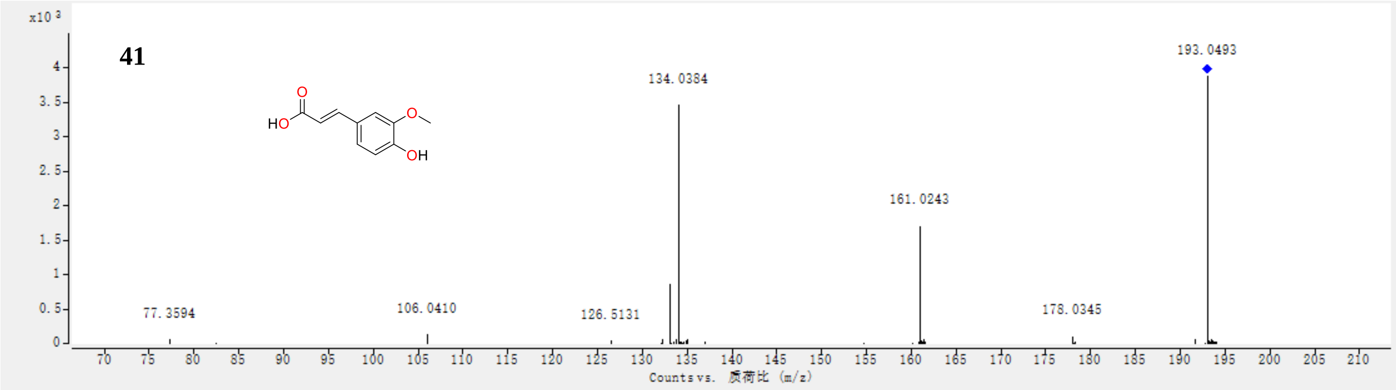
41
x10 3
193. 0493
134. 0384
161. 0243
77. 3594
106. 0410
126. 5131
178. 0345
HO
O
OH
Counts vs. 质荷比 (m/z)

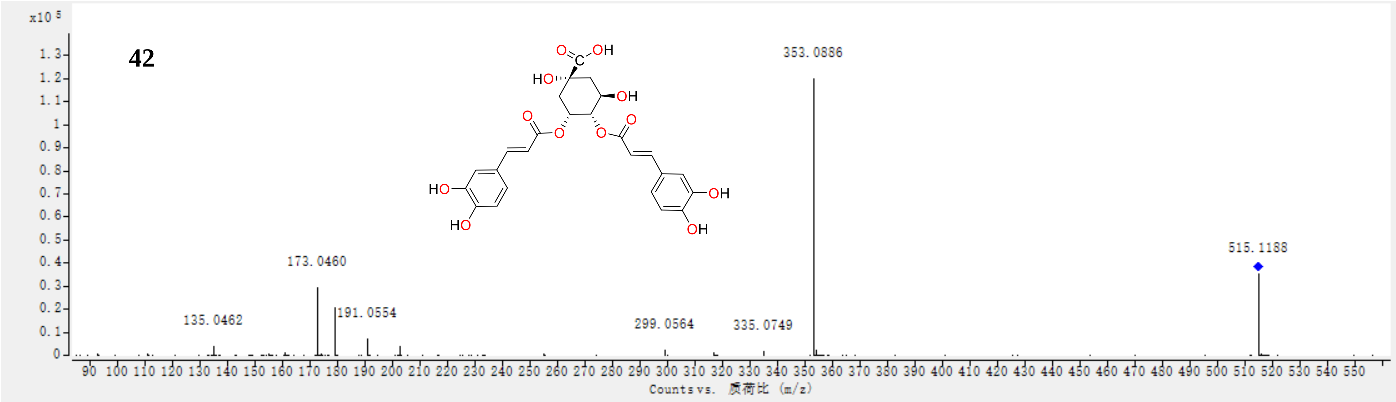
42
x10 5
353. 0886
515. 1188
173. 0460
135. 0462
191. 0554
299. 0564
335. 0749
HO
OH
Counts vs. 质荷比 (m/z)

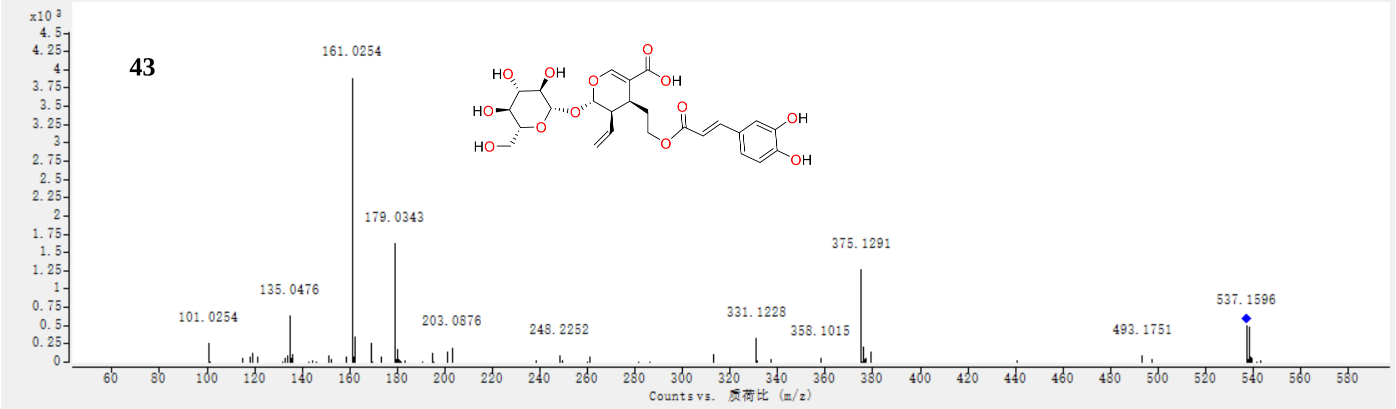
43
x10 3
161. 0254
179. 0343
375. 1291
135. 0476
101. 0254
203. 0876
248. 2252
331. 1228
358. 1015
493. 1751
537. 1596
HO
OH
Counts vs. 质荷比 (m/z)

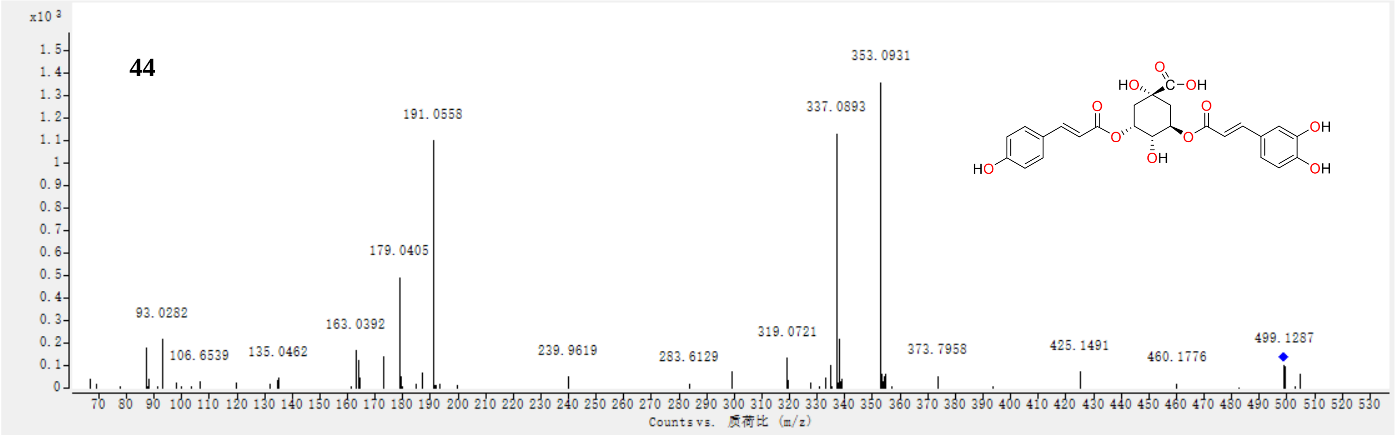
44
x10 3
353. 0931
337. 0893
191. 0558
179. 0405
93. 0282
106. 6539
135. 0462
163. 0392
239. 9619
283. 6129
319. 0721
373. 7958
425. 1491
460. 1776
499. 1287
HO
OH
Counts vs. 质荷比 (m/z)

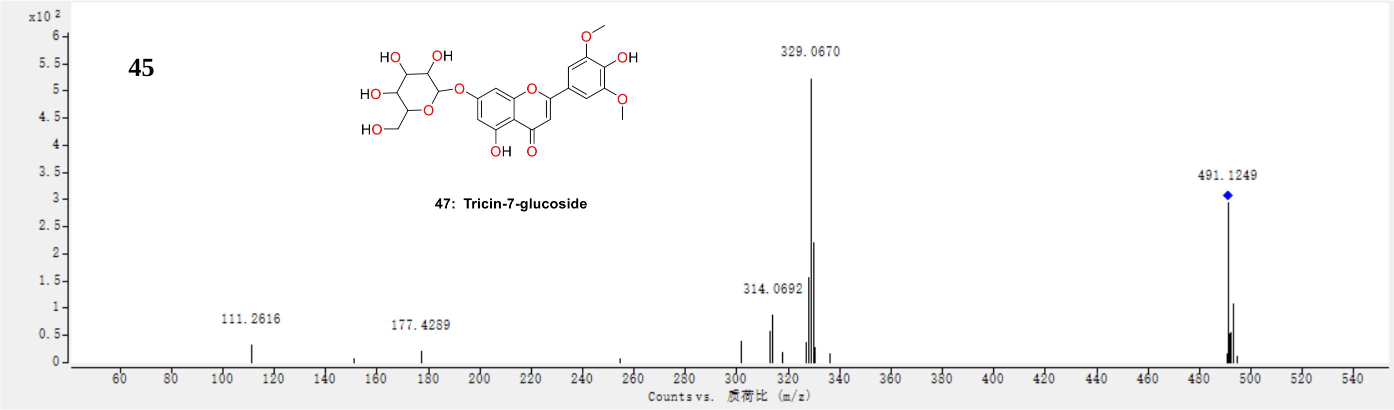
x10 2
45
47: Tricin-7-glucoside
329. 0670
491. 1249
314. 0692
111. 2616
177. 4289
Counts vs. 质荷比 (m/z)

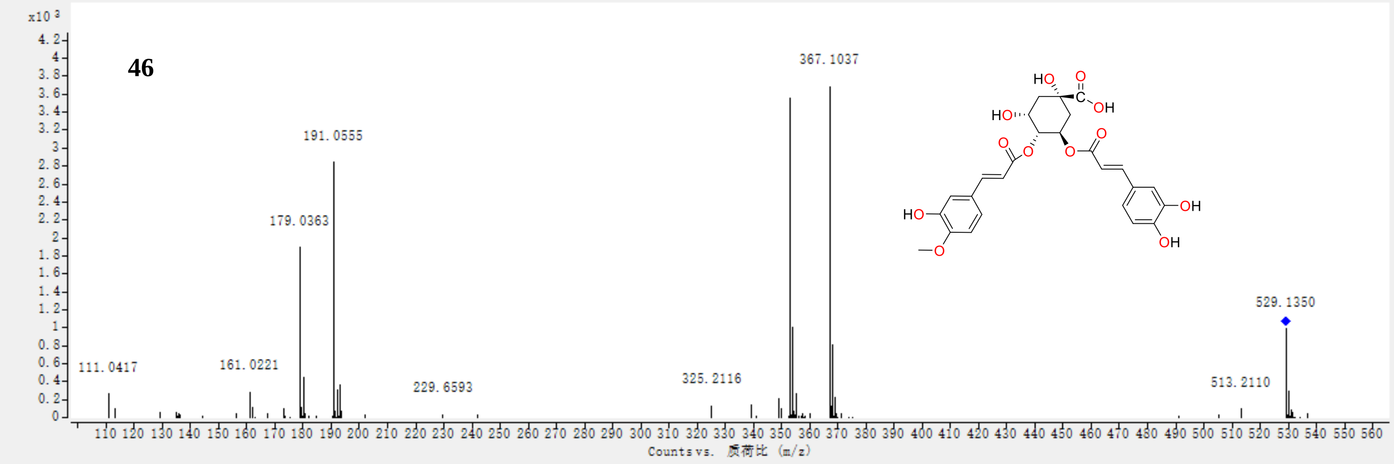
x10 3
46
367. 1037
191. 0555
179. 0363
529. 1350
111. 0417
161. 0221
229. 6593
325. 2116
513. 2110
Counts vs. 质荷比 (m/z)

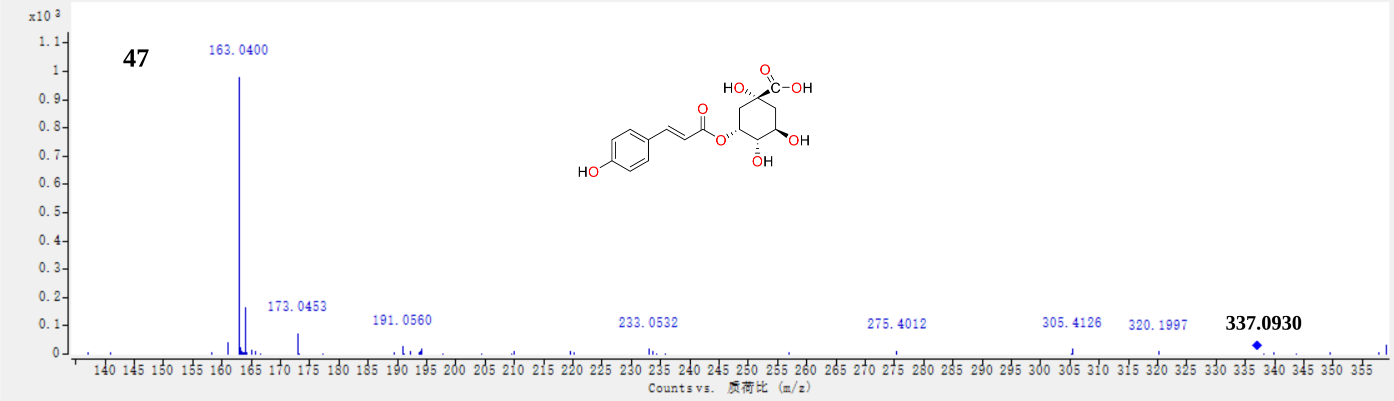
x10 3
47
163. 0400
173. 0453
191. 0560
233. 0532
275. 4012
305. 4126
320. 1997
337.0930
Counts vs. 质荷比 (m/z)

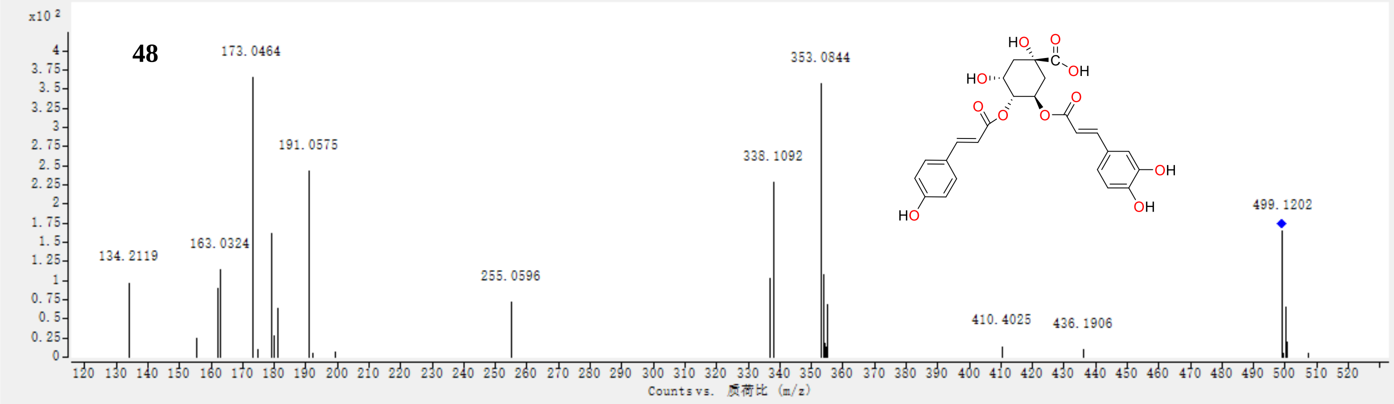
x10 2
48
173. 0464
353. 0844
191. 0575
338. 1092
134. 2119
163. 0324
255. 0596
499. 1202
410. 4025
436. 1906
Counts vs. 质荷比 (m/z)

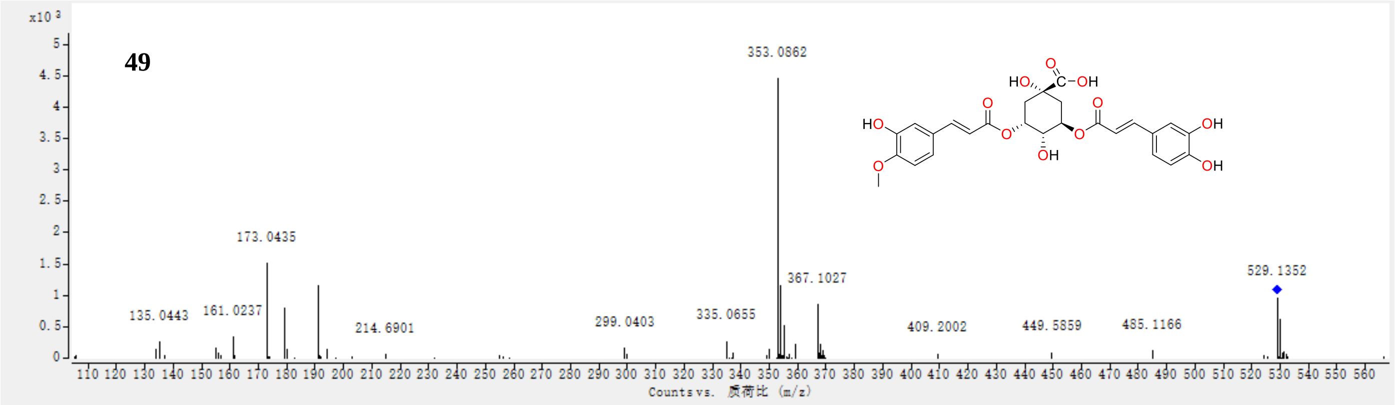
49
x10 3
353. 0862
173. 0435
135. 0443
161. 0237
214. 6901
299. 0403
335. 0655
367. 1027
409. 2002
449. 5859
485. 1166
529. 1352
Counts vs. 质荷比 (m/z)

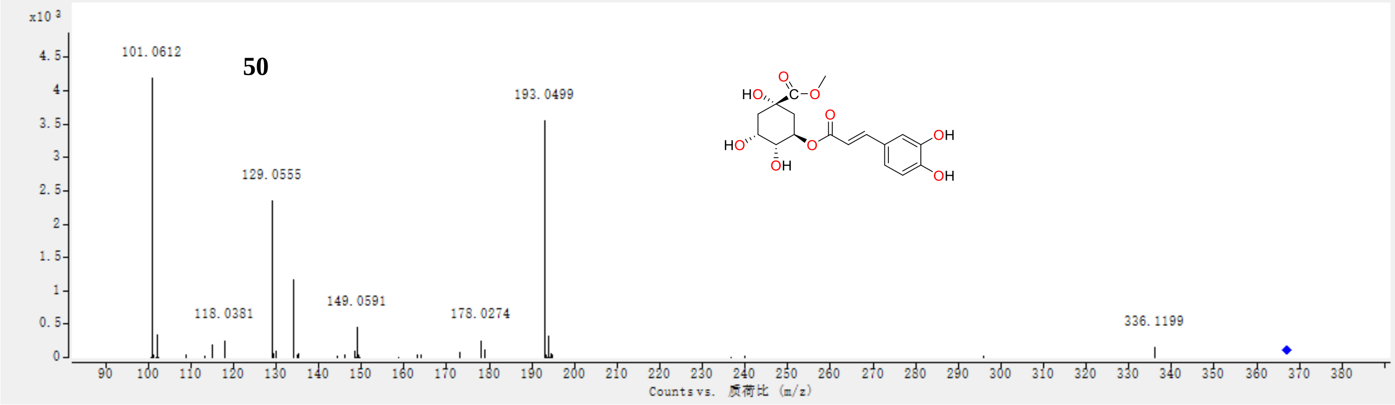
50
x10 3
101. 0612
193. 0499
129. 0555
118. 0381
149. 0591
178. 0274
336. 1199
Counts vs. 质荷比 (m/z)

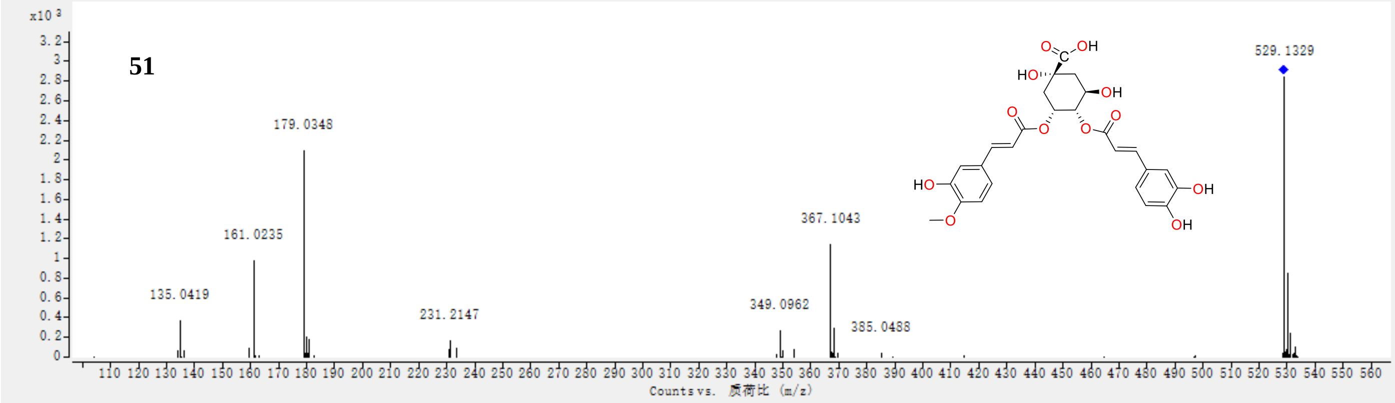
51
x10 3
529. 1329
179. 0348
161. 0235
135. 0419
231. 2147
349. 0962
367. 1043
385. 0488
Counts vs. 质荷比 (m/z)

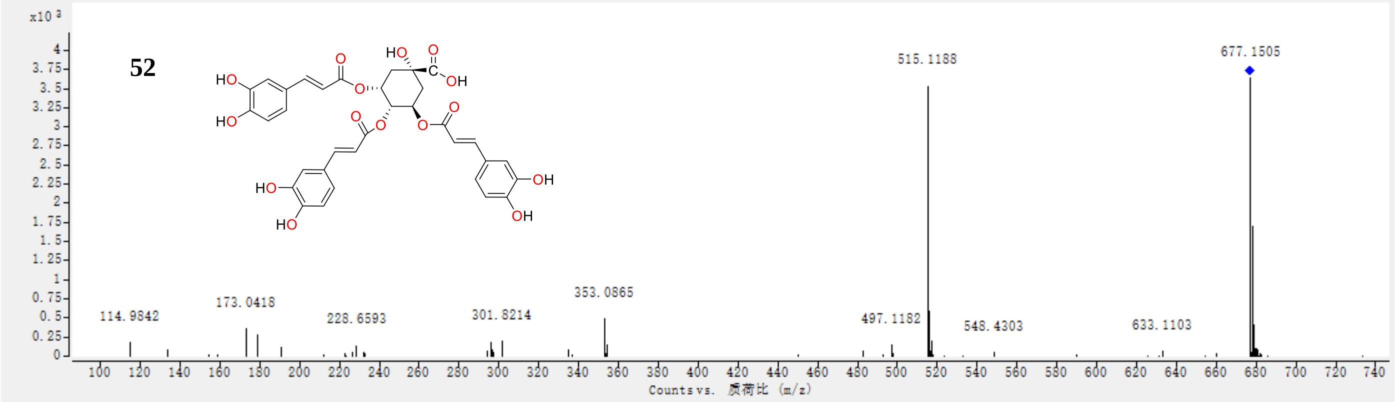
52
x10 3
515. 1188
677. 1505
114. 9842
173. 0418
228. 6593
301. 8214
353. 0865
497. 1182
548. 4303
633. 1103
Counts vs. 质荷比 (m/z)

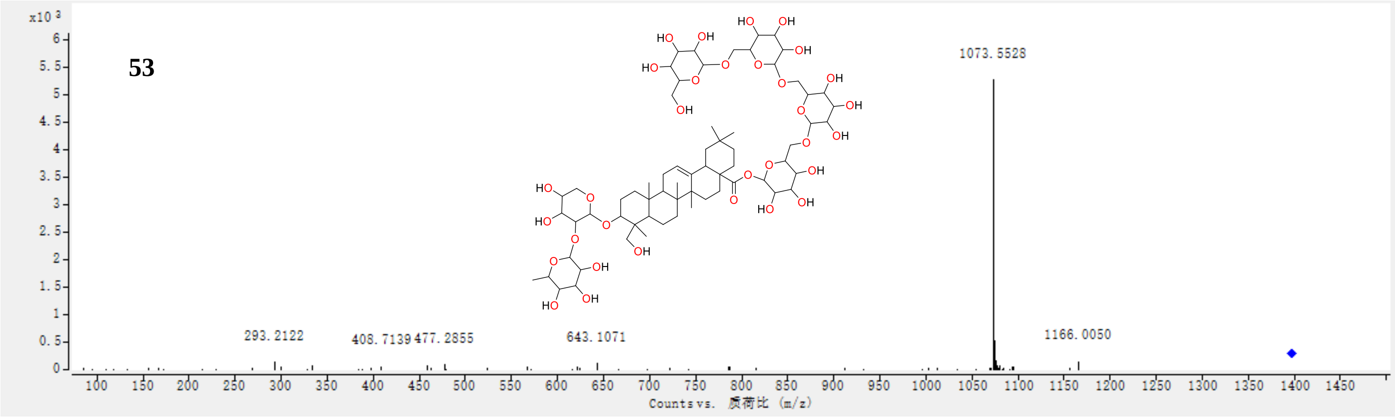
x10 3
53
1073. 5528
293. 2122
408. 7139
477. 2855
643. 1071
1166. 0050
Counts vs. 质荷比 (m/z)

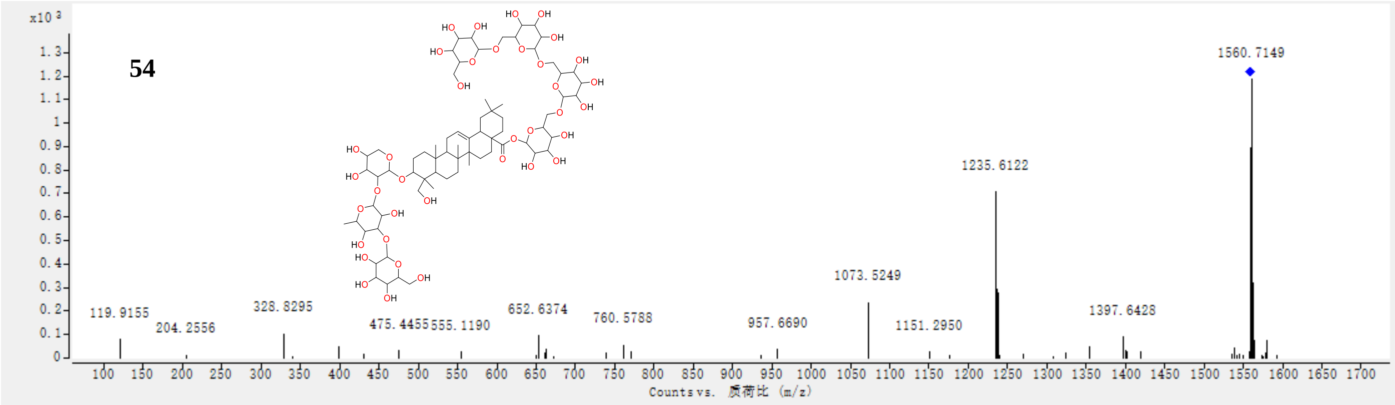
x10 3
54
1560. 7149
1235. 6122
1073. 5249
119. 9155
204. 2556
328. 8295
475. 4455
555. 1190
652. 6374
760. 5788
957. 6690
1151. 2950
1397. 6428
Counts vs. 质荷比 (m/z)

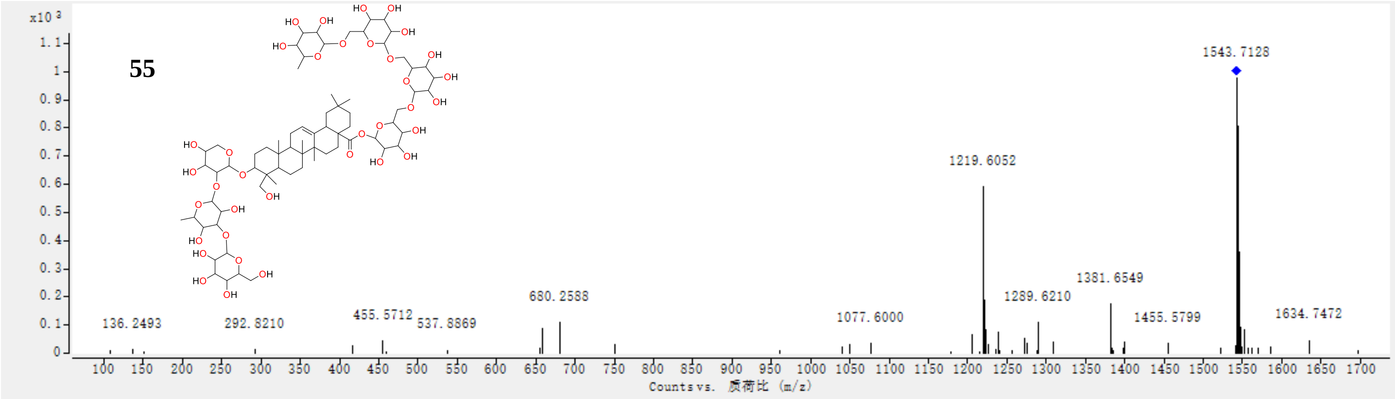
x10 3
55
1543. 7128
1219. 6052
1381. 6549
1289. 6210
680. 2588
136. 2493
292. 8210
455. 5712
537. 8869
1077. 6000
1455. 5799
1634. 7472
Counts vs. 质荷比 (m/z)

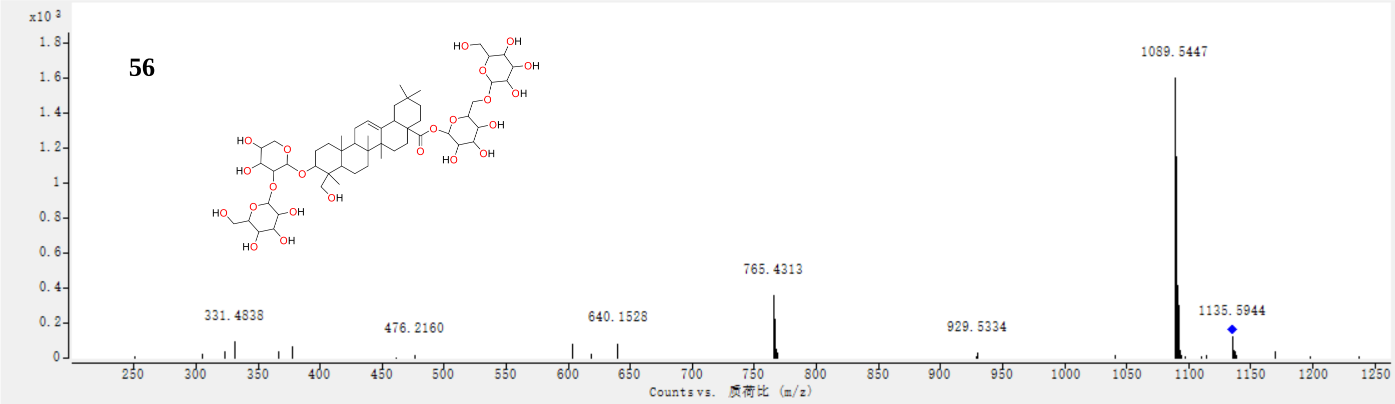
x10 3
56
1089. 5447
765. 4313
331. 4838
476. 2160
640. 1528
929. 5334
1135. 5944
Counts vs. 质荷比 (m/z)

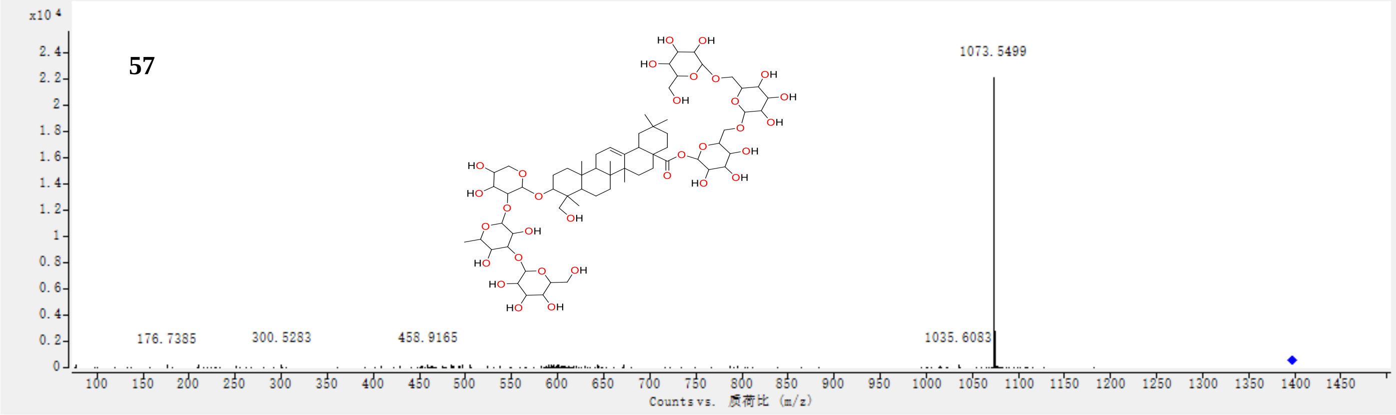
57
x10 4
1073.5499
176.7385
300.5283
458.9165
1035.6083
Counts vs. 质荷比 (m/z)

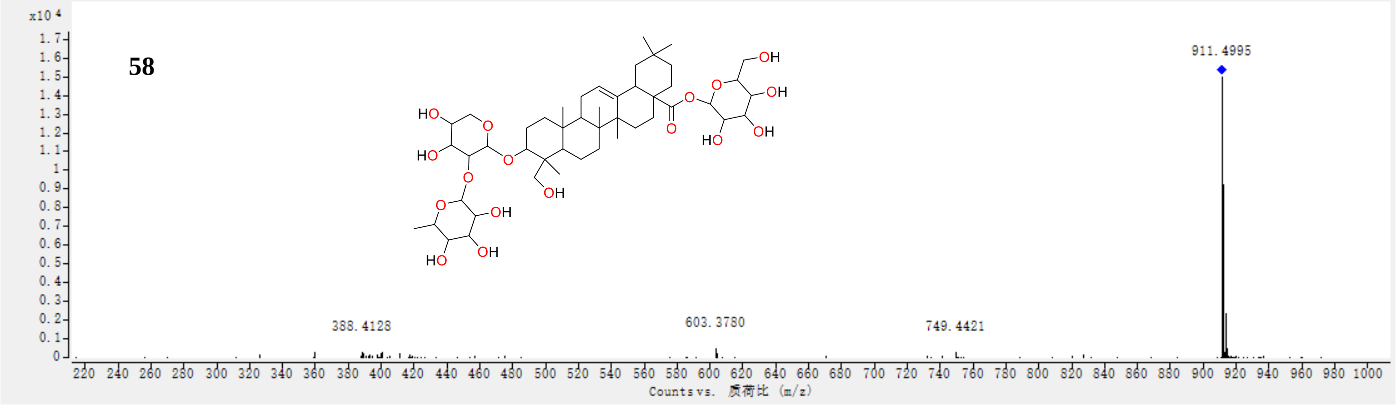
58
x10 4
911.4995
388.4128
603.3780
749.4421
Counts vs. 质荷比 (m/z)

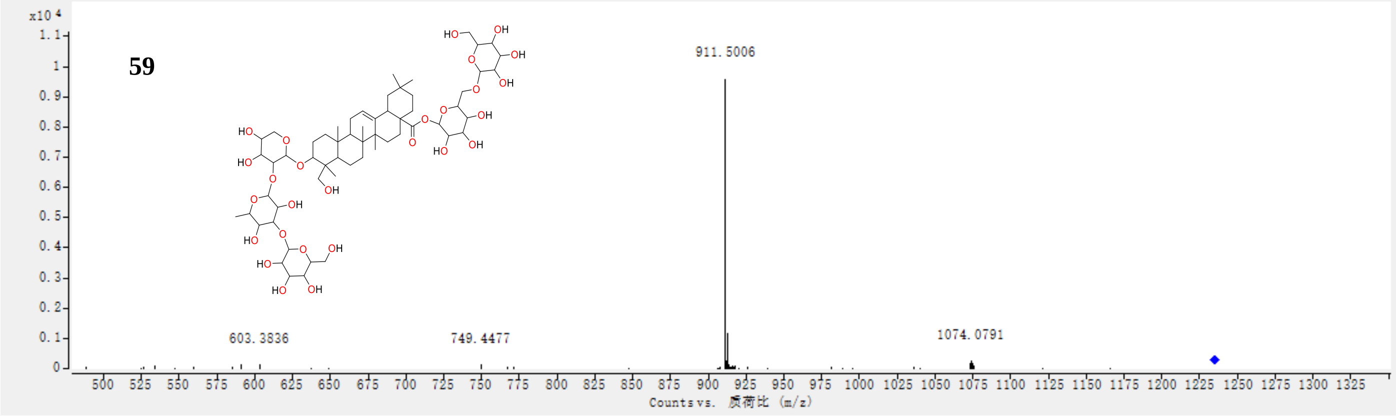
59
x10 4
911.5006
603.3836
749.4477
1074.0791
Counts vs. 质荷比 (m/z)

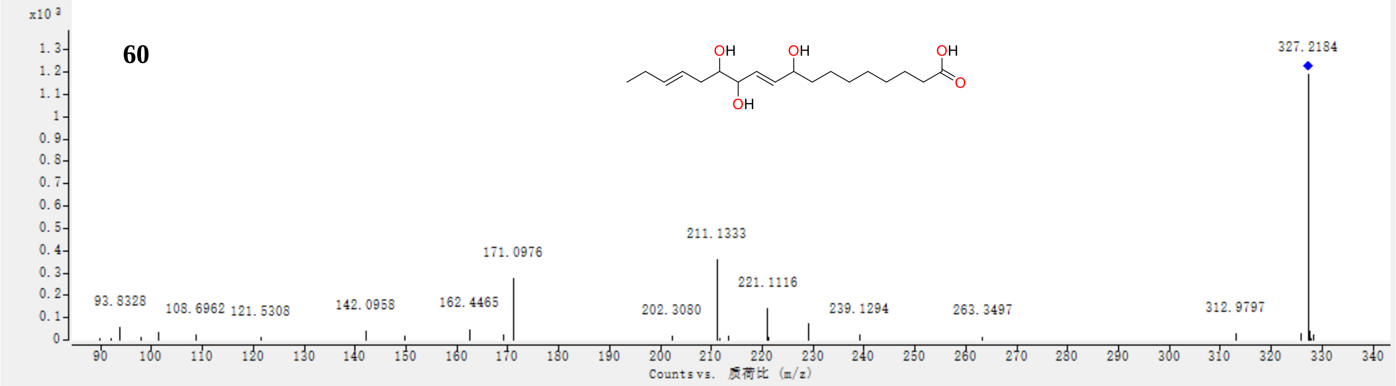
60
x10 3
327.2184
211.1333
171.0976
221.1116
93.8328
108.6962
121.5308
142.0958
162.4465
202.3080
239.1294
263.3497
312.9797
Counts vs. 质荷比 (m/z)

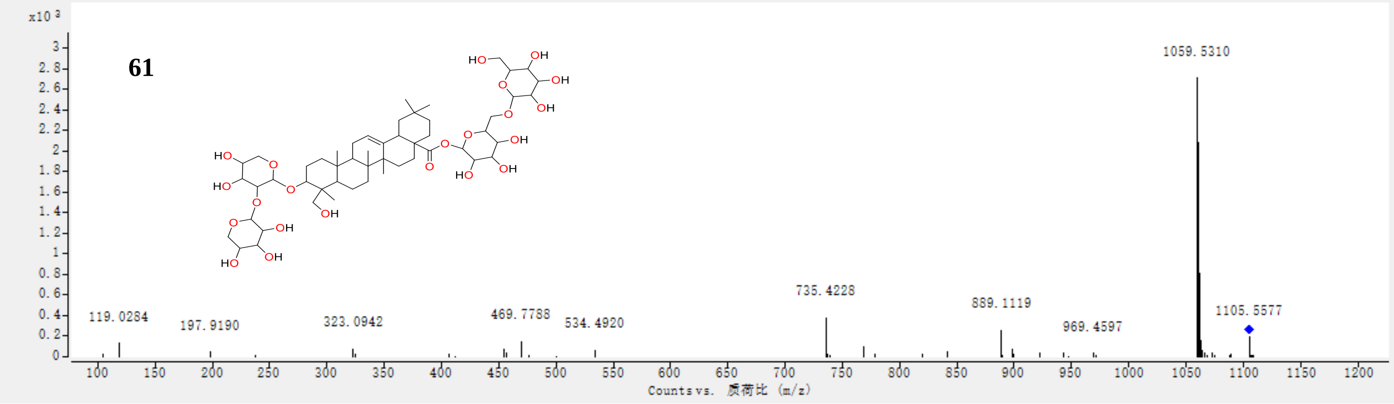
61
x10 3
119.0284
197.9190
323.0942
469.7788
534.4920
735.4228
889.1119
969.4597
1059.5310
1105.5577
Counts vs. 质荷比 (m/z)

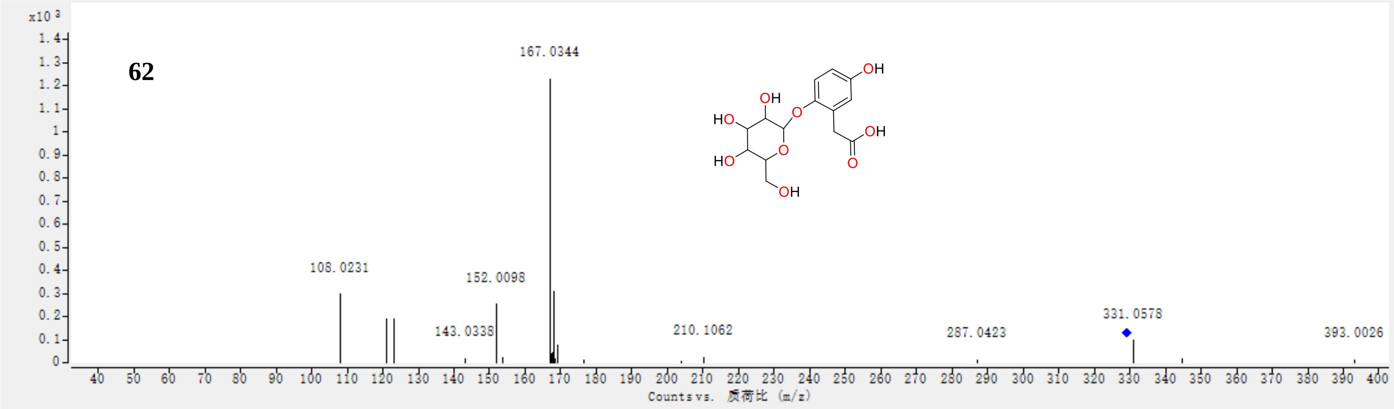
62
x10 3
108.0231
143.0338
152.0098
167.0344
210.1062
287.0423
331.0578
393.0026
Counts vs. 质荷比 (m/z)

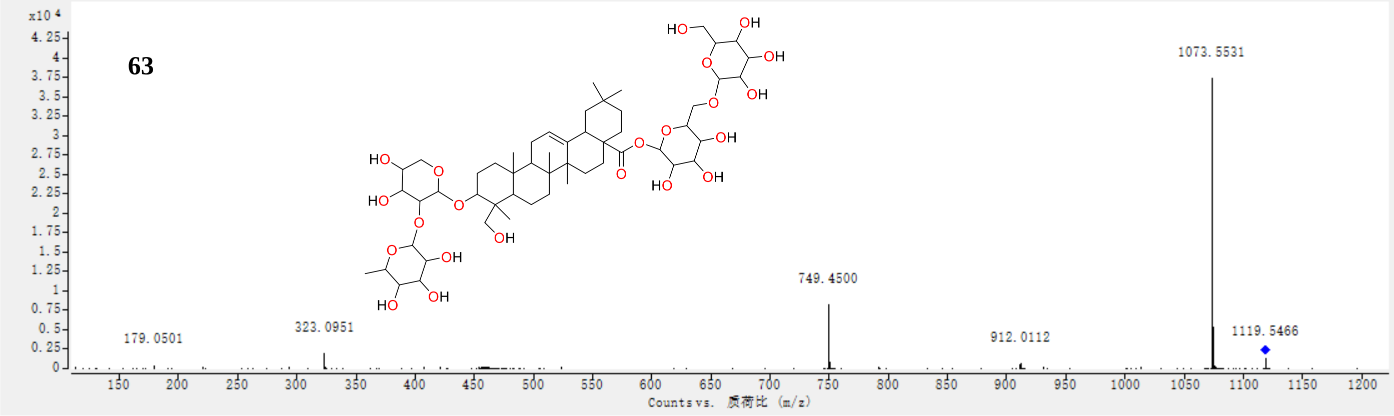
63
x10 4
179.0501
323.0951
749.4500
912.0112
1073.5531
1119.5466
Counts vs. 质荷比 (m/z)

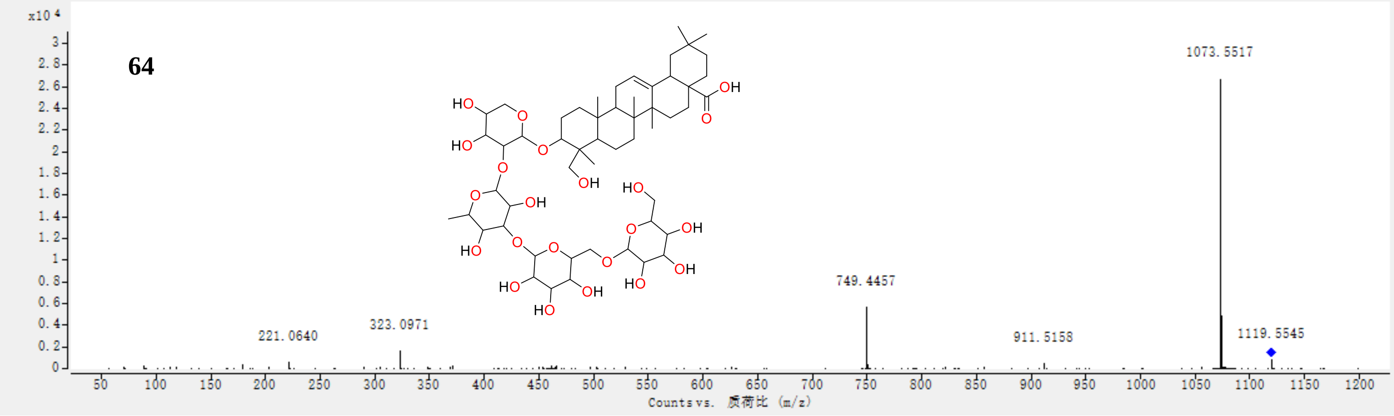
64
x10 4
221.0640
323.0971
749.4457
911.5158
1073.5517
1119.5545
Counts vs. 质荷比 (m/z)

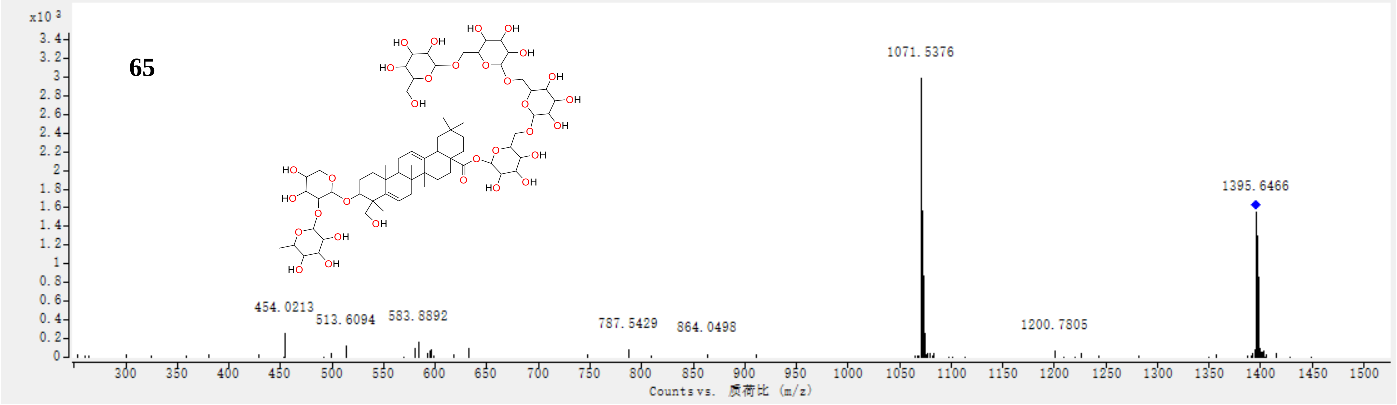
65
x10 3
1071. 5376
1395. 6466
454. 0213
513. 6094
583. 8892
787. 5429
864. 0498
1200. 7805
Counts vs. 质荷比 (m/z)

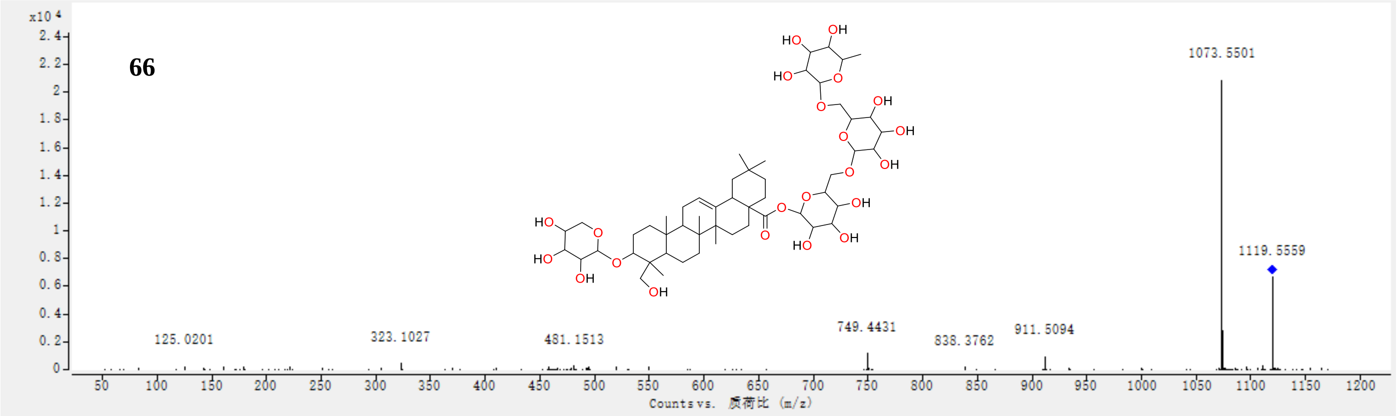
66
x10 4
1073. 5501
1119. 5559
125. 0201
323. 1027
481. 1513
749. 4431
838. 3762
911. 5094
Counts vs. 质荷比 (m/z)

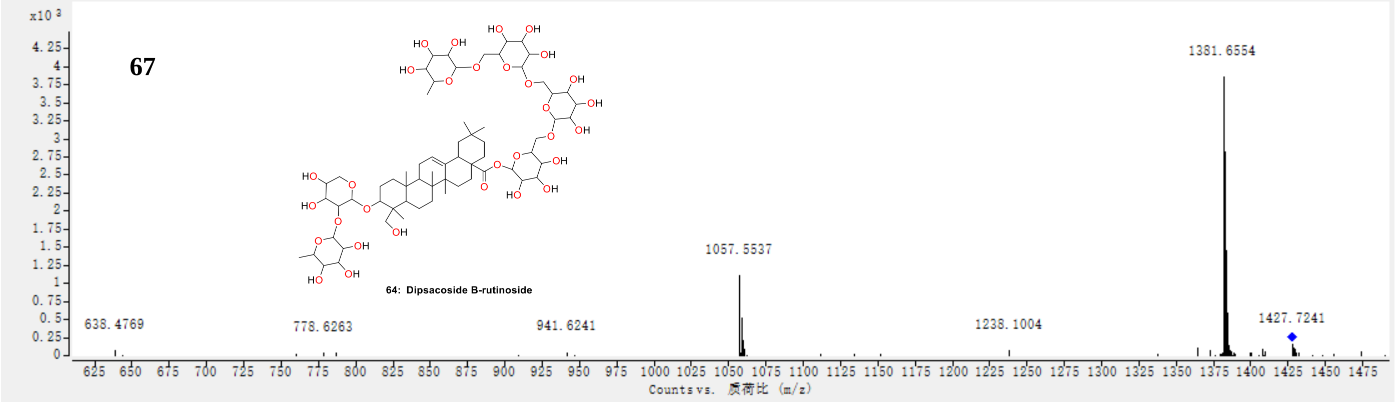
67
x10 3
1381. 6554
1057. 5537
638. 4769
778. 6263
941. 6241
1238. 1004
1427. 7241
64: Dipsacoside B-rutinoside
Counts vs. 质荷比 (m/z)

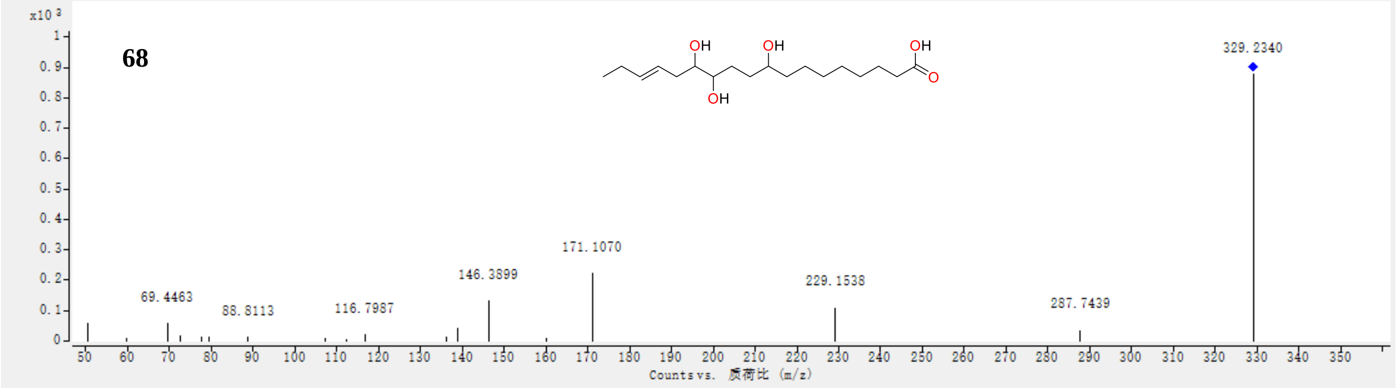
68
x10 3
329. 2340
171. 1070
146. 3899
229. 1538
69. 4463
88. 8113
116. 7987
287. 7439
Counts vs. 质荷比 (m/z)

69

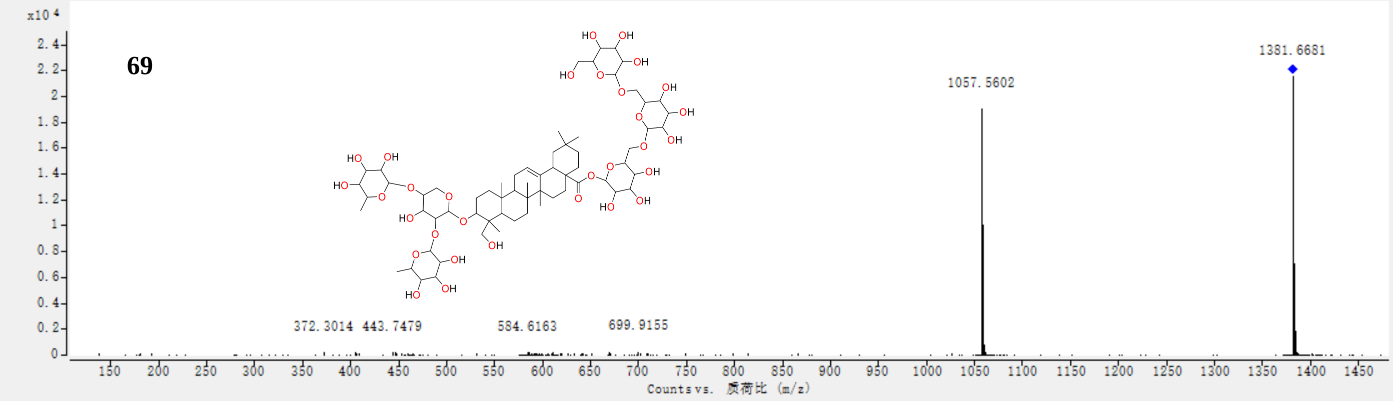
x10 4
1381.6681
1057.5602
372.3014
443.7479
584.6163
699.9155
Counts vs. 质荷比 (m/z)

70

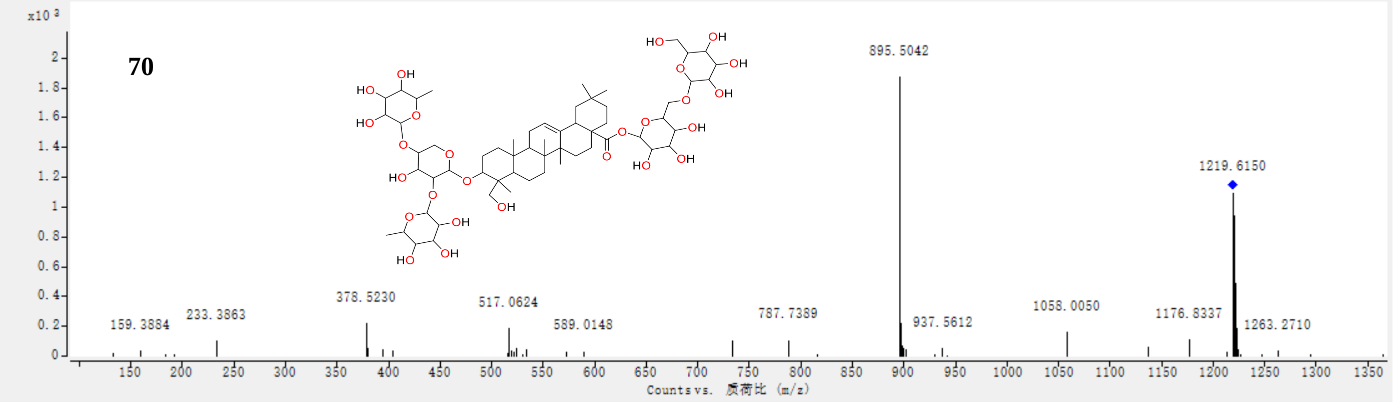
x10 3
895.5042
1219.6150
159.3884
233.3863
378.5230
517.0624
589.0148
787.7389
937.5612
1058.0050
1176.8337
1263.2710
Counts vs. 质荷比 (m/z)

71

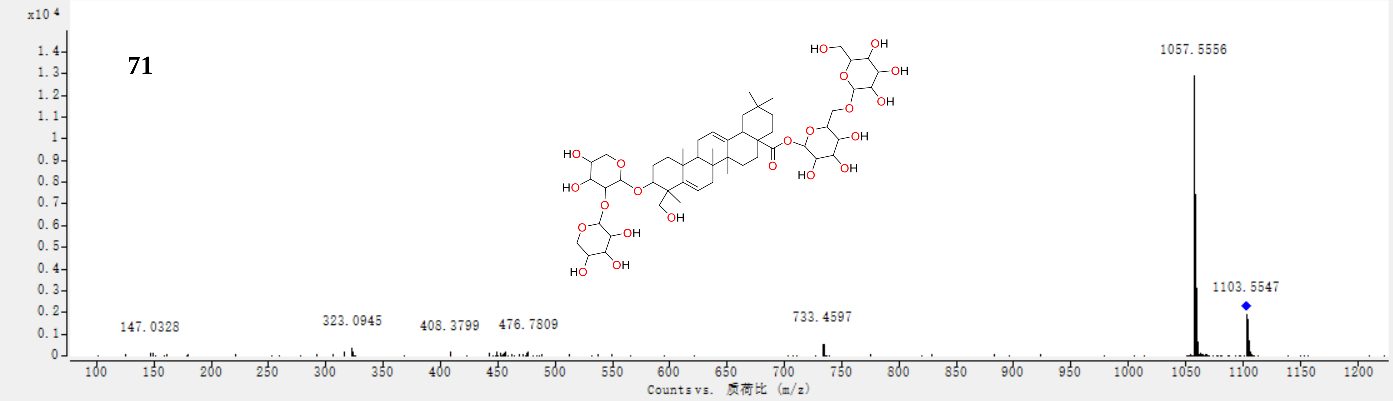
x10 4
1057.5556
1103.5547
147.0328
323.0945
408.3799
476.7809
733.4597
Counts vs. 质荷比 (m/z)

72

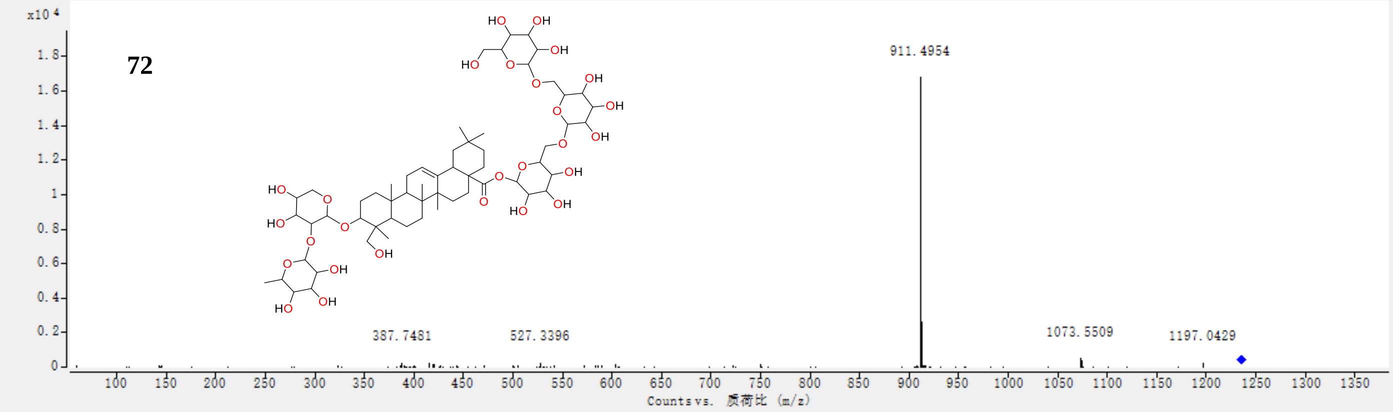
x10 4
911.4954
387.7481
527.3396
1073.5509
1197.0429
Counts vs. 质荷比 (m/z)

Table S1 the components of high fat diet

Component	Proportion (%)
Lard	31.6
Casein	25.8
Maltodextrin	16.1
Saccharose	8.9
Cellulose	6.5
Composite minerals	5.8
Soybean oil	3.2
Multivitamin	1.3
L-cystine	0.4
Choline bitartrate	0.3

Table S2 The groups, number of mice and dose design of anti-diabetic experiment for *L. japonica* buds, *L. hypoglauca* buds, roots, stems, and leaves extracts.

Groups	Number of Mice	Dose (mg/kg)
NC	10	--
MC	10	--
Acarbose	10	30
LJ-B	10	200
LH-B	10	200
LH-R	10	200
LH-S	10	200
LH-L	10	200

Table S3 The groups, number of mice and dose design of anti-diabetic experiment for seven compounds

Group	Number of Mice	Dose (mg/kg)
NC	10	--
MC	10	--
Acarbose	10	30
NE-A	10	30
CH-A	10	30
CR-A	10	30
SE	10	30
IA-A	10	30
IA-B	10	30
IA-C	10	30
